# Texture‐Taste Interactions: Exploring the Effect of Thickener Concentration on the Sensory Perception of Sweet, Sour, and Salty Tastes

**DOI:** 10.1111/jtxs.70042

**Published:** 2025-09-21

**Authors:** Gunalan Dhamodharan, James Makame, Alissa A. Nolden

**Affiliations:** ^1^ Department of Food Science University of Massachusetts Amherst Massachusetts USA

**Keywords:** dysphagia, rheology, taste perception, thickened liquids, viscosity

## Abstract

The addition of thickeners to beverages remains the primary strategy for managing dysphagia. However, thickened beverages have poor compliance among patients, primarily due to a dislike of the taste and flavor. While thickeners produce a higher viscosity, which helps reduce the risk of aspiration, there is evidence that they also reduce taste sensations. Previous research suggests that rheological properties might explain the impact on taste perception; however, recent findings do not demonstrate this interaction. Therefore, this study aims to explore the impact of Nestle ThickenUp Clear on the taste intensity of sucrose (sweet), citric acid (sour), and NaCl (salty) when mixed at three concentrations compared to an unthickened control. The samples were analyzed for rheological properties (including viscosity and tan delta). A total of 56 untrained healthy participants completed the Temporal Check All That Apply (TCATA) and rated taste intensity and liking. This study presented significant findings on physical textural properties and sensory aspects; most importantly, Linear Mixed Models (LMM) revealed that saltiness (*β* = −4.16), sourness (*β* = −3.29), and sweetness (*β* = −3.43) all decreased significantly with the addition of thickener (*p* < 0.001), with the magnitude and slope varying considerably for each tastant. However, viscosity did not result in a decrease in taste intensity (as indicated by a lower marginal *R* ^2^ than conditional *R* ^2^). These findings emphasize that unique interactions between tastants and thickeners drive taste perception, rather than being solely determined by physical rheological measures (such as viscosity and viscoelasticity). Future studies can extend these findings to other thickeners and more complex beverages, which will aid in identifying effective strategies to improve the taste and flavor profiles of thickened beverages.

## Graphical abstract

Thickened beverages reduce the risk of aspiration but negatively impact taste perception. This study found that adding xanthan‐gum‐based commercial thickener significantly decreased sweetness, sourness, and saltiness perception, though viscosity and tribology did not explain the reduction in taste ratings. Reduced taste intensity appears to be driven by thickener–tastant interactions rather than rheological or textural properties.

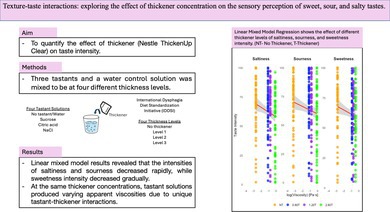

## 1 Introduction

The texture of food and beverages has a direct impact on taste perception, both physiologically and physically (Pangborn et al. 1973). This research area is crucial for enhancing safe swallowing in individuals with dysphagia, a swallowing disorder that affects an estimated 15 million people in the United States, leading to a higher risk of malnutrition and dehydration (Hong et al. 2024). Due to the swallowing difficulties associated with this disorder, individuals face significant challenges in having safe eating and drinking experiences (Whelan 2001; Logemann 1998). Adding hydrocolloids to foods and beverages alters their physiological texture by increasing viscosity, which slows down bolus velocity and improves pharyngeal transit time, thus helping to prevent aspiration during swallowing (Stokes et al. 2013; Reimers‐Neils et al. 1994). Despite this, many patients express dissatisfaction with thickened liquids due to their undesirable texture, taste, and flavor, which can decreased compliance and inadequate nutritional intake (McCurtin et al. 2018; García‐Almeida et al. 2025). Hence, it is essential to understand how the addition of thickeners affects taste perception to improve acceptance and well‐being in managing dysphagia. There is limited research on the impact of commercial thickeners used in managing dysphagia on sensory attributes and taste perception, highlighting the need for more studies in this field. Therefore, enhancing our understanding of taste‐texture interactions will improve the scientific framework and lead to innovative solutions that enhance sensory experiences and consumer acceptance.

Rheology serves as a fundamental tool in evaluating the physical texture parameters of foods and beverages, traditionally used to predict mouthfeel, how a food or beverage is perceived in the mouth (Stokes et al. 2013). One common textural measurement is viscosity, which refers to a liquid's internal resistance to flow (Viswanath et al. 2007). Physically, variations in viscosity can affect taste perception in numerous ways. Although findings vary regarding whether increasing viscosity enhances or diminishes taste perception, the prevailing opinion is that enhanced viscosity generally reduces taste perception (Hollowood et al. 2002; Malone et al. 2003; Cook et al. 2002; Vaisey et al. 1969; Moskowitz and Arabie 1970; Pangborn et al. 1973; Christensen 1980; Baines and Morris 1988). One reason for the diminished taste response is that hydrocolloids hinder the interaction of tastants with taste receptors (Cook et al. 2002). This theory is supported by the concept of critical overlap concentration (c*), which indicates the point where viscosity sharply increases as thickener concentration rises, marking the point at which hydrocolloid chains begin to overlap and restrict molecular movement (Baines and Morris 1988). Many studies have investigated c* as a significant measurement for identifying the concentration that leads to decreased taste perception (Baines and Morris 1988; Cook et al. 2002; Han et al. 2014; Hollowood et al. 2002; Koliandris et al. 2010; Malone et al. 2003; Wagoner et al. 2018). Cook et al. (2002) demonstrated that incorporating hydroxypropyl methylcellulose (HPMC) into individual solutions containing sucrose, NaCl, citric acid (CA), and quinine HCl above the critical c* concentration notably reduced sweetness and saltiness, but did not significantly alter sourness or bitterness. It was noted that the sweetness intensity of sucrose decreased in the presence of guar gum, whereas no change occurred when mixed with λ‐carrageenan; conversely, the sweetness of aspartame exhibited the opposite trend above c* (Cook et al. 2002). These findings demonstrate that the relationship between physical properties and taste is intricate and influenced by various factors, including the specific tastant and thickener used (Garcia et al. 2005).

Thus, the purpose of this study is to explore the sensory properties of a commonly used xanthan gum‐based thickener (Nestle ThickenUp Clear) and investigate its influence on the three tastes—sweetness of sucrose, sourness of citric acid, and saltiness of NaCl. This study aims to quantify the effects of different tastants on rheological properties and investigate their relationship with taste perception across varying thickener concentrations. Overall, this approach helps to inform the complex interplay between taste perception and textural properties. The findings of this research may lead to an improved understanding of how adding a xanthan gum‐based thickener affects taste perception related to specific tastants, enabling the design of a safe‐swallowing product that does not compromise sensory taste and texture.

## 2 Materials and Methods

### 2.1 Sample Preparation

Food‐grade tastants, including sucrose (≥ 99.5% Sigma Aldrich, USA), CA (≥ 99% Sigma Aldrich, USA), and NaCl (≥ 99.5% Sigma Aldrich, Canada), were used. The xanthan gum‐based thickener Nestle Resource ThickenUp Clear (TUC), purchased from Nestle Healthcare Nutrition, USA, was used to prepare the thickened liquids. The thickener has a composition (% w/w) of xanthan gum (33%), maltodextrin (MD) (66.4%), and potassium chloride (0.6%), as reported by Gamonpilas et al. (2023).

Sixteen thickened solutions were prepared (Table 1). The concentrations represent three different IDDSI levels (1–3) selected from Gamonpilas et al. (2023). This resulted in the following thickener concentrations (w/v%): 0.80T, 1.20T, and 2.60T, representing the IDDSI levels 1, 2, and 3, respectively. The formulations were designed to maintain a constant concentration of the tastant (sucrose 0.5, CA 0.056, and NaCl 0.3 M). The concentrations of the tastants were selected based on previous literature (Spinelli et al. 2024; Sato et al. 2022) and underwent pilot testing. Briefly, the thickened liquids were prepared by preparing a stock solution for each tastant, adding tastants to 30 mL of distilled water (density: 0.99 g/mL), and the tastant solution was mixed in a magnetic stirrer at 100–150 RPM for 5 mins. Then, the thickener was added slowly while the solution was continuously stirred at low RPM to prevent particle aggregation. Once added, the thickened liquids were mixed in a magnetic stirrer at 150–250 RPM for 30 min. This method aligns with manufacturers' directions and prior studies (Rofes et al. 2014; Kongjaroen et al. 2022). The samples were prepared freshly each day, and measurements were performed within 30 min after preparation, held at room temperature.

**TABLE 1:** Formulation and sample codes (NT: no thickener; T: contains thickener).

S. No	Composition	Sample code	Tastant conc. (M)	Thickener conc. (%w/v)
1	Water	Water‐NT	—	—
2	Water + Thickener	Water‐0.80T	—	0.80
3	Water + Thickener	Water‐1.20T	—	1.20
4	Water + Thickener	Water‐2.60T	—	2.60
5	Sucrose	Sucrose‐NT	0.5	—
6	Sucrose + Thickener	Sucrose‐0.80T	0.5	0.80
7	Sucrose + Thickener	Sucrose‐1.20T	0.5	1.20
8	Sucrose + Thickener	Sucrose‐2.60T	0.5	2.60
9	Citric acid	CA‐NT	0.056	—
10	Citric acid + Thickener	CA‐0.80T	0.056	0.80
11	Citric acid + Thickener	CA‐1.20T	0.056	1.20
12	Citric acid + Thickener	CA‐2.60T	0.056	2.60
13	NaCl	NaCl‐NT	0.3	—
14	NaCl + Thickener	NaCl‐0.80T	0.3	0.80
15	NaCl + Thickener	NaCl‐1.20T	0.3	1.20
16	NaCl + Thickener	NaCl‐2.60T	0.3	2.60

### 2.2 Rheological Analysis

#### 2.2.1 Apparent Shear Viscosity

The apparent viscosity of the samples was measured using a Discovery Hybrid Rheometer HR‐20 (TA Instruments, USA) equipped with DIN concentric cylinders and an Aluminum Peltier plate—65,308. Briefly, the viscosity flow analysis is as follows: 23 mL of the sample was measured and added to the concentric cylinders with the geometry gap set to 5908.64 μm (fixed for all samples). Before the measurement, the samples were pre‐sheared for 120 s at a 0.0001 s^−1^ shear rate to remove any shear history acquired during sample loading, and the temperature of the samples was equilibrated to 37°C to simulate in‐mouth oral temperature (Pangborn et al. 1970). Then, the apparent shear viscosity was determined over the shear rate range of 0.1–1000 s^−1^ with steady‐state sensing, an axial force of 1 N, 10 points per decade, and a sensitivity of 0.1, respectively. Duplicates were performed. The power law model, defined as Equation (1), fitted to investigate the relationship between the shear viscosity and shear rate.

(1) $$\sigma =\kappa \cdot \dot{\;{\left(\gamma \right)}^{n}}$$

In Power law, where *σ* is the shear stress (Pa), ($\dot{\gamma }$) is the shear rate (S^−1^), n is the flow index, and *κ* is the consistency co‐efficient (Pa s^*n*^).

#### 2.2.2 Viscoelasticity

The viscoelasticity of the samples was examined using a frequency amplitude shear sweep test with the same geometry (concentric cylinders) and experimental procedures used to determine viscosity (same geometry gap and sample volume). Initially, a strain amplitude shear sweep test was performed to determine the Linear Viscosity Region (LVR) for the different types of formulations, using a strain range of 0.1%–100% with steady‐state sensing at a fixed angular frequency of 10 rad/s at 37°C, with an axial force of 1 N. Then, the frequency sweep test was performed from 0.1 rad/s to 100 rad/s at 37°C with a strain range of 0.1%–1%, all within the LVR region, using a fresh sample for each test to determine the elastic and viscous properties. The logarithmic sweep was performed with a 1 N axial force, 0.1 sensitivity, and 10 points per decade. Then, the storage moduli G′ and loss moduli G′′ were recorded, and the experiment was repeated in duplicate. The tan (*δ*) was calculated by dividing the loss moduli G′′ by the storage moduli G′.

### 2.3 Sensory Evaluation

#### 2.3.1 Participants

Individuals for the sensory study were recruited from the area surrounding the University of Massachusetts, Amherst, through posters and online advertisements. All interested individuals were screened for the following inclusion criteria: aged between 18 and 45 years; nonpregnant or nursing; nonsmokers; without lip, cheek, or tongue piercings; no loss of taste or smell; no cold, flu, or fever symptoms; no history of swallowing difficulties (dysphagia); or food allergies. A total of 56 participants (22 males and 36 females) took part in the study, with a mean age of 24 (±4). The study protocols received approval from the Institutional Review Board at the University of Massachusetts (protocol number 5472), and individuals provided consent prior to participation.

#### 2.3.2 Stimuli Preparation

On the day of testing, thickened liquids were prepared 1 h before the session, following the sample preparation methodology outlined in the instrumentation section. The only adjustment made was to stir the samples in a magnetic stirrer for 5 min. This change was necessary due to the large number of stimuli, which prevented alterations in the characterization of xanthan gum dissolution.

#### 2.3.3 Study Protocol

Participants were instructed not to eat or drink for at least 1 h before the sensory evaluation. Each test session lasted 30 min, and all evaluations were conducted using Compusense software (Version 24.0.29869, 2024/11/13) from Compusense Inc., Ontario, Canada. Stimuli were presented at room temperature in 10 mL aliquots, served in clear plastic medicine cups. The order of the samples was fully randomized using the Williams Latin Square design method, and each stimulus was labeled with a three‐digit code. A 30‐s break was enforced between stimuli, and participants were instructed to rinse with water. The study began with a questionnaire in which participants reported their hunger and thirst levels, as well as the timing of their last meal. After this, they became familiar with the evaluation method and learned how to use the scales effectively. The sensory evaluation was performed in the following order: (i) Temporal‐Check All That Apply (TCATA), (ii) taste intensity, and (iii) overall liking.

#### 2.3.4 Temporal Check‐All‐That‐Apply (TCATA)

TCATA is a method in which participants report the attributes they experience over time. This study selected nine attributes based on previous literature utilizing TCATA methods (Sharma et al. 2022; Brouwer et al. 2024) and pilot testing. Participants received a brief description to familiarize themselves with each attribute (Table 2). After this task, participants were instructed to take a full sample into their mouths and press the “start” button. First, they were asked to swirl the sample for 15 s, checking all perceived attributes, not just the most dominant one. After 15 s, participants were instructed to swallow and continue selecting attributes until reaching 60 s. The selected attributes faded automatically to minimize cognitive load, as outlined by Ares et al. (2016). Each participant's order of attributes remained constant within a session, while the presentation order varied across participants (Brouwer et al. 2024).

**TABLE 2:** List of sensory attributes and definitions used for TCATA evaluation.

Definition	
Attribute	Taste category
Salty	The salty taste intensity was associated with sodium chloride/salt.
Sour	The sour taste intensity was associated with citric acid/lemon.
Sweet	The sweet taste intensity associated with sucrose/sugar.
Mouthfeel category	
Thick	This is related to the viscosity of the sample, how hard/easy (amount of effort needed) it is to move the sample in the mouth while orally processing it.
Mouthcoating	The amount of film on the mouth surfaces, the coating on the oral surfaces inside the mouth.
Thin	Product readily flows, related to the viscosity of the sample (the opposite of thick).
Slippery/slick	The slippery feel of the sample while the sample is being moved from the front to the back of the mouth.
Cohesive	Tendency of the product mass to stay together in one piece.
Smooth	The sensation of smoothness when the tongue slides the sample across the roof of the mouth.

#### 2.3.5 Rating Scales: Taste Intensity and Overall Liking

To assess taste intensity, a 100‐point generalized visual analog scale (gVAS) developed by Hayes et al. (2013) and Kershaw and Running (2019) was used to measure the intensity of sensations, including sweetness, sourness, and saltiness. All three taste intensity scales were presented for each stimulus. The gVAS included two anchors: “No Sensation” at (0) and “Strongest Sensation Ever Experienced” at (100), with no intervals. To familiarize participants with the scale and ensure comprehension, simplified warm‐up questions based on Kershaw and Running (2019) were incorporated during the instructional phase. Responses were analyzed, and any participants who misused the scale were excluded based on the criteria set by Kershaw and Running (2019). For overall liking, participants' responses were collected on a bipolar hedonic scale (Bartoshuk et al. 2005). This scale featured three intervals: −100 for the strongest dislike, 0 for neutral, and 100 for the strongest liking, with no additional intervals.

### 2.4 Statistical Analysis

#### 2.4.1 Instrumental

The raw data for rheological properties were obtained using TRIOS Software from TA Instruments, USA. The Excel Solver add‐in (Microsoft Excel version 16.96.1) was utilized over a shear rate range of 0.1–1000 s^−1^ for the power law model fitting. Then, the two‐way ANOVA was performed with a significance level of *p* < 0.01. The mean values and standard deviations were calculated from the experimental replicates for the viscosity flow curve and tan delta plots. The results were visualized using the multcompview, rstatix, ggplot, patchwork, dplyr, emmeans, and agricolae packages with R statistical software (version 4.4.2), R Core Team (2024).

#### 2.4.2 Sensory

Participants were screened based on their responses to the gVAS warm‐up questions, with 56 qualified. The responses of these qualified participants were analyzed using R software (Version: 4.4.2, R Core Team 2024). TCATA data analysis was conducted following the methodology detailed by Brouwer et al. (2024) and Sharma et al. (2022) to assess the temporal sensory perception of various samples. TCATA curves were generated for each sample (*n* = 16) per attribute based on individual responses at specific time points using the tempR package (Castura 2024). The chance level and significance level were calculated using the method detailed by Pineau et al. (2009) and the rstatix package. The significance level line indicates that the attribute is consistent at the panel level, while the chance level represents attributes considered inconsistently cited or insignificant. Data smoothing was applied through cubic spline smoothing (smoothing parameter = 0.5) to reduce noise (Meyners and Castura 2018). Subsequently, TCATA parameters—AUC (Area Under the Curve, representing the cumulative area beneath the TCATA curve), Tmax (the time of maximum citation), Cmax (the maximum citation proportion of the TCATA curve), Cend (citation proportion at the final time point of the TCATA curve), and T0.5 (the time required for the maximum citation proportion to decline to half of its peak)—were extracted for each attribute per sample per interval. For both Cmax and Cend, significant differences (*p* < 0.05) were assessed via Fisher's pairwise comparison test as described by Brouwer et al. (2024), while AUC significance differences (*p* < 0.05) were analyzed using the lme4 package. No statistical analysis was conducted for T0.5 and Tmax, as those values were derived from average citation proportions. All comparisons of TCATA parameters were conducted within tastant groups, rather than across all 16 samples, as different groups contained different tastants, making comparisons across tastant groups irrelevant.

The taste intensity distribution plot was analyzed, focusing on the sourness intensity determined solely from CA samples (CA‐NT, 0.80T, 1.20T, 2.60T), sweetness from sucrose samples (sucrose‐NT, 0.80T, 1.20T, 2.60T), and salt intensity from NaCl samples (NaCl‐NT, 0.80T, 1.20T, 2.60T) to explore variations in taste intensity with viscosity. A one‐way ANOVA and Tukey's HSD analyses were conducted to identify significant differences. Taste intensity plots for samples without added tastants (Water‐NT, 0.80T, 1.20T, 2.60T), deemed tasteless by Park et al. (2012), were excluded based on consistent findings from both the pilot and main sensory studies. However, overall liking included all samples (sucrose, CA, NaCl) with control groups (samples without added tastants). A two‐way ANOVA and Tukey's HSD analyses were performed to identify significant differences.

Next, Linear Mixed Models (LMM) were employed, which effectively analyze datasets with nonindependent observations, such as repeated measurements or nested experimental designs (Galecki and Burzykowski 2013; Bates et al. 2015). These models separate variance into fixed effects (population‐level predictors) and random effects (group‐ or individual‐level variability) to facilitate accurate inference, while addressing autocorrelation and heteroscedasticity (Bates et al. 2015; West et al. 2014). Models were fitted using Restricted Maximum Likelihood (REML) to obtain unbiased estimates for random effects (Bates et al. 2015). Fixed effects were determined through backward elimination, with nonsignificant terms (*p* > 0.05) removed iteratively while comparing model fit using the Akaike Information Criterion (AIC). Random effects, such as participant‐specific intercepts, were retained if their inclusion significantly improved model fit, assessed through likelihood ratio tests (LRTs) (West et al. 2014). An unstructured covariance matrix was initially assumed for repeated‐measures data to maximize flexibility in modeling correlations. Simpler structures (e.g., compound symmetry, autoregressive) were tested if convergence issues arose, with the final structure chosen based on AIC (West et al. 2014). The significance of fixed effects was assessed using Satterthwaite's approximation for degrees of freedom, producing *t*‐statistics and *p*‐values (Kuznetsova et al. 2017). Post hoc pairwise comparisons adjusted for multiple testing utilized the Sidak method. Model assumptions were validated using normality, homoscedasticity, and influential outlier metrics, including Cook's distance and leverage. All analyses were conducted in R version 4.4.2 using the lme4 package (Bates et al. 2015) for model fitting and the lmerTest (Kuznetsova et al. 2017) for hypothesis testing. Data visualization was created using ggplot2, and model diagnostics were employed using the performance package. Full model summaries, pairwise contrasts, and ANOVA results are found in Supporting Tables S1–S4. All statistical analyses for sensory evaluations were performed using the ggplot2, rstatix, lme4, nlme, lmerTest, performance, multcomp, emmeans, dplyr, and ggrepel packages in R, along with standard error bars with R statistical software (version 4.4.3, R Core Team 2024).

## 3 Results and Discussion

### 3.1 Physical Textural Measures

The apparent viscosity of the samples at a shear rate of 50 s^−1^, defined as *η*_50_, along with the fitting parameters for the power models, is reported in Table 3. As expected, the apparent viscosity of the sample increased as the concentration of thickener (ThickenUp Clear) increased. The *η*_50_ across thickener concentrations (NT, 0.80T, 1.20T, 2.60T) is found to be higher for tastant solutions than for the control (no added tastant), due to molecular interactions with the thickening medium (mechanisms are discussed below). Notably, at higher thickener concentrations (2.60% w/v), the *η*_50_ for NaCl is higher than for CA, sucrose, or control liquids.

**TABLE 3:** Summary of the power law model fitting.

Sample	*η*_50_ (Pa s)	*k* (s)	*n*	*R* ^2^
Water‐0.80T	0.10054ᵈ	0.477 ± 0.003ᶠ	0.602 ± 0.002ᵇ	0.969 ± 0.02
Water‐1.20T	0.17788ᶜ	1.354 ± 0.008ᵉ	0.481 ± 0.003ᶜ	0.997 ± 0.00
Water‐2.60T	0.32026ᵇ	9.052 ± 0.045ᶜ	0.146 ± 0.002ᶠ	0.998 ± 0.00
Sucrose‐0.80T	0.12572ᶜᵈ	0.695 ± 0.004ᶠ	0.562 ± 0.002ᵇ	0.982 ± 0.01
Sucrose‐1.20T	0.19004ᶜ	1.711 ± 0.008ᵉ	0.439 ± 0.003ᶜ	0.992 ± 0.00
Sucrose‐2.60T	0.36813ᵇ	10.52 ± 0.06ᵇ	0.142 ± 0.002ᶠ	0.998 ± 0.00
CA‐0.80T	0.07306ᵈ	0.404 ± 0.003ᶠ	0.562 ± 0.002ᵇ	0.986 ± 0.01
CA‐1.20T	0.15100ᶜ	1.357 ± 0.007ᵉ	0.439 ± 0.003ᶜ	0.999 ± 0.00
CA‐2.60T	0.74386ᵃ	15.27 ± 0.08ᵇ	0.250 ± 0.003ᵉ	0.999 ± 0.00
NaCl‐0.80T	0.08150ᵈ	0.760 ± 0.006ᶠ	0.430 ± 0.002ᶜ	0.999 ± 0.00
NaCl‐1.20T	0.16571ᶜ	2.500 ± 0.012ᵈ	0.308 ± 0.003ᵈ	0.998 ± 0.00
NaCl‐2.60T	0.80781ᵃ	16.63 ± 0.09ᵃ	0.208 ± 0.003ᵉ	0.998 ± 0.00

*Note:* A one‐way ANOVA was performed; different lower‐case letters in the same columns indicate that samples significantly differ (*p* < 0.05).

The power law model exhibited an excellent fit for most samples (*R* ^2^ = 0.993 ± 0.01), except for those without added thickener, which were excluded (Table 3). This poor fit for non‐thickened samples is attributed to the instrument's limitations in measuring samples with very low viscosity, as previously reported by Gamonpilas et al. (2023) and Gupta et al. (2016). A larger *K* value suggests that the shear thinning transition occurs at lower shear rates. All samples showed increased *K* values as the thickener concentration increased, suggesting that the shear‐thinning behavior shifted to higher shear rates (i.e., 2.60t > 1.20t > 0.80T). This can be explained by the enhanced entanglement of xanthan gum chains at higher concentrations, resulting in stronger polymer–polymer interactions and, consequently, greater resistance to flow (Morris et al. 1981). Therefore, higher thickener concentrations resulted in an earlier onset of shear‐thinning behavior and more pronounced viscosity reduction, reflected in higher *K* values. Overall, the power law model suggests that as thickener concentration increases, both *η*₅₀ and *K* values rise, resulting in a shift in shear‐thinning behavior toward lower shear rates, which aligns with prior work (Badia‐Olmos et al. 2022; Gamonpilas et al. 2023; Kongjaroen et al. 2022).

**FIGURE 1:**
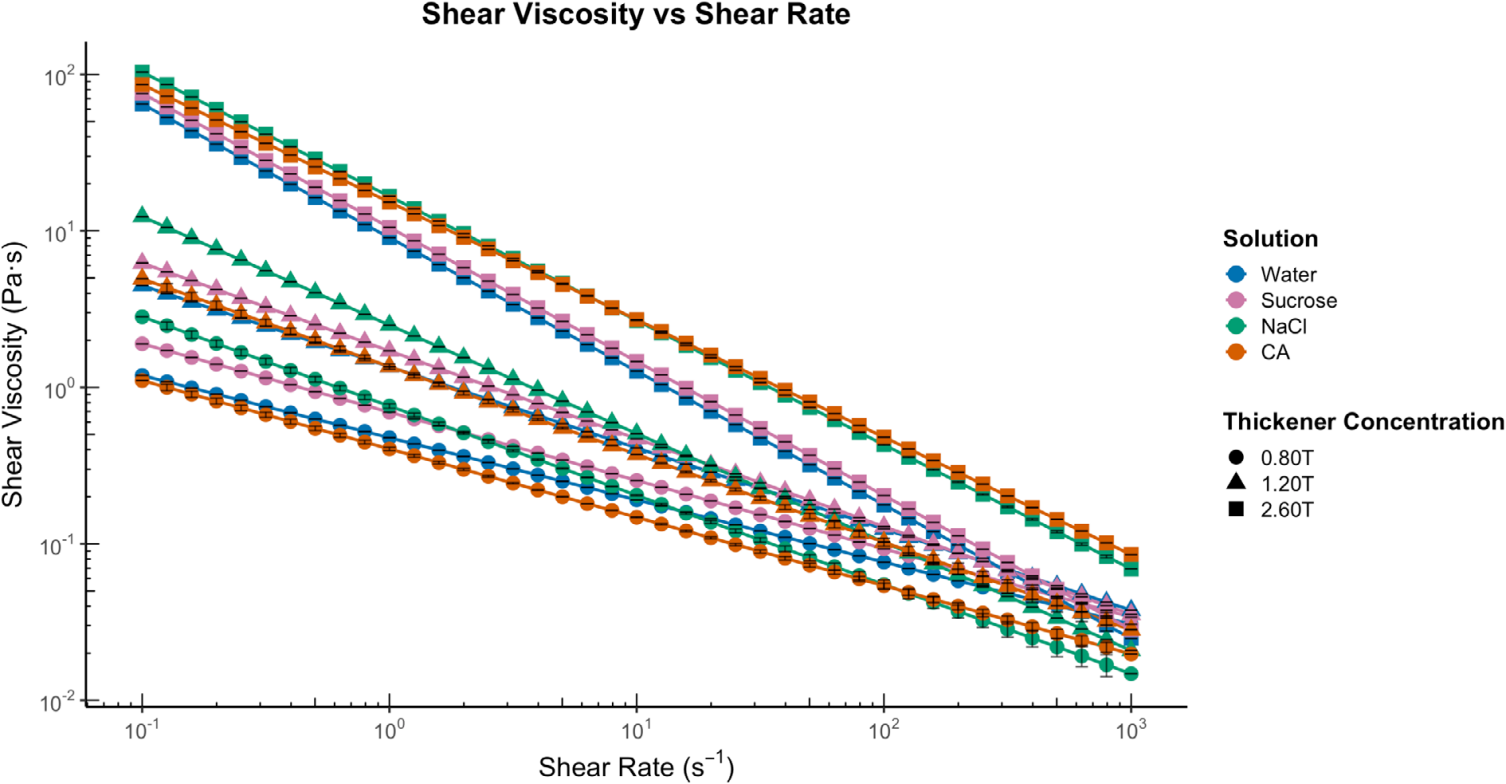
Combined flow curves of all samples showing the effects of thickener concentrations across tastants.

Flow properties are important parameters to measure in dysphagic foods, as they affect the flow behavior of the bolus during swallowing, such as pharyngeal transit time (Newman et al. 2016). A recent study by Ferris et al. (2021) showed that flow timing measures (significantly affected by bolus conditions such as volume and viscosity) are potential markers for swallowing, along with distinction pressure and luminal opening. Figure 1 reports the flow properties of the different thickened liquids. The samples with added thickener displayed concentration‐dependent shear‐thinning behavior, driven by the increase in polymer concentration, which raises the number of entanglements (Wyatt and Liberatore 2009). Notably, sucrose solutions exhibited a slower degree of shear thinning compared to the NaCl and CA solutions at 0.80% (w/v) and 1.20% (w/v) thickener concentrations. However, contrasting results were found for sucrose‐2.60T. The reason for the sucrose‐2.60T solution's faster shear thinning behaviors is the active competition between the polar groups in the xanthan gum and sucrose for water, which limits the availability of water for xanthan gum and reduces polymer–solvent interaction (Bak and Yoo 2023, 2018; Doyle et al. 2006; Khouryieh et al. 2007). In contrast, the CA solutions exhibited altered flow behavior characterized by a reduced degree of shear thinning. Particularly, the CA‐2.60T solution can be attributed to the charge screening effect, resulting in an ordered conformation due to an increase in pH (Bueno and Petri 2014). However, our study does not clarify whether the pH level was substantial enough to alter the structure of xanthan.

All NaCl solutions exhibit low shear‐thinning behavior (high *n* value) due to the addition of NaCl, which may relate to a slimy sensation in thickened liquids (Cho et al. 2015). The charge screening effect explains the increased viscosity of NaCl across all concentrations. Xanthan gum in aqueous solutions typically exhibits an ordered single or double helix conformation or a disordered coil conformation. At 25°C in water, it exists in a disordered state, forming a random, extended helix due to hydrogen bonding and electrostatic repulsion (Norton et al. 1984). The weak hydrogen bonding among xanthan chains breaks under shear. Introducing NaCl creates a helical conformation as the charged trisaccharide collapses, transitioning from a disordered to an ordered state (Norton et al. 1984; Rochefort and Middleman 1987; Muller et al. 1986). In this ordered state, xanthan chains become compact, resulting in a reduced hydrodynamic size that is dependent on the salt concentration. Norton et al. (1984) indicated that xanthan chains stabilize into a helical structure at NaCl concentrations above 40 mM. Here, the 0.3 M NaCl used is relatively high. NaCl solutions at 1.20T and 2.60T showed higher shear viscosity than sucrose or CA solutions. Increasing the thickener concentration likely enhances the shear viscosity in NaCl solutions. Cho et al. (2015) similarly found that 0.3% w/w (0.05 M) NaCl with xanthan gum yielded higher viscosity than 0% and 0.5% (0.10 M) NaCl, attributing this to synergistic effects. They noted that higher NaCl concentrations (> 0.3% w/w) led to reduced viscosity. However, the synergistic interaction mechanism between NaCl and xanthan gum remains unclear, necessitating further research on their concentration and molecular interactions.

**FIGURE 2:**
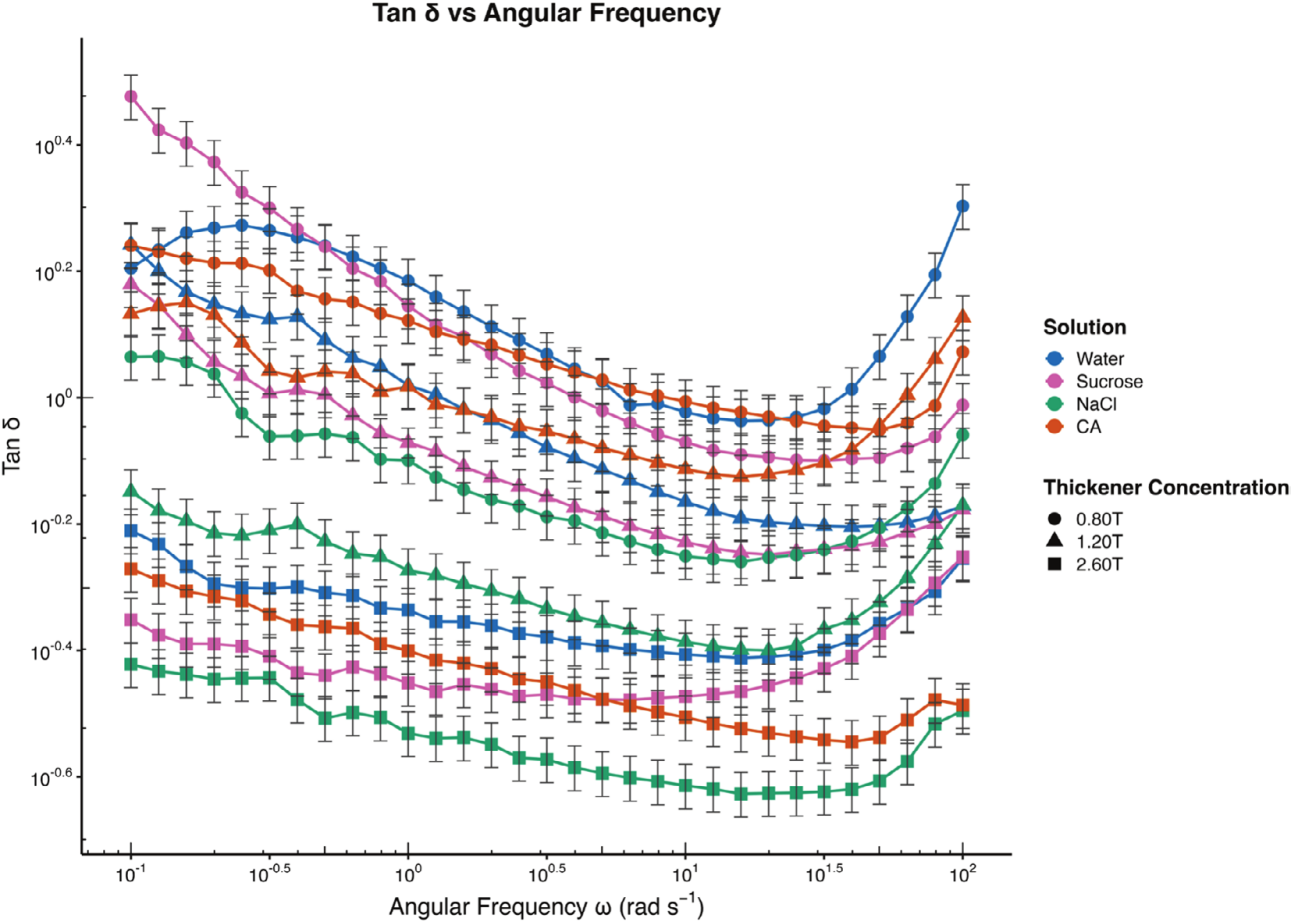
Combined Tan (delta) curves across all samples, showing the effects of tastant solutions on varying thickener concentrations.

To interpret the viscoelasticity properties, the loss tangent plots were employed, which is a measure of the ratio of G′′ to G′ (the higher the tan *δ*, the higher the energy dissipation). Numerous studies have correlated higher elastic properties and strong shear thinning of a liquid with pleasant swallowing (Funami 2011; Ishihara et al. 2011; Kim et al. 2017; Vieira et al. 2020). In general, a decrease in tan (delta) was observed with increasing thickener concentration (Figure 2), exhibiting the characteristic behavior of xanthan gum and xanthan gum‐based thickener, as reported in previous studies (Cho et al. 2015; Jo and Yoo 2019). This behavior is attributed to the formation of more complex junctions, resulting in stronger network formations, as explained by Gamonpilas et al. (2023). For all the sample groups (sucrose, CA, NaCl, and water), the molecular mobility occurred at lower frequencies due to the rapid entanglement of polymers, followed by a relatively stable energy dissipation and higher molecular mobility at higher frequencies. This nonlinear behavior suggests a structural transition in the material throughout the measured frequency, unique to the formation of the tastant‐thickener network. In the presence of thickener, NaCl decreased the tan *δ* values across all thickener concentrations (0.80T, 1.20T, and 2.60T) for the measured frequency range due to the self‐induced aggregation effect (Jo and Yoo 2019). Specifically, NaCl‐2.60T is highly crosslinked compared to CA‐2.60T and sucrose‐2.60T, with the thickener matrices due to the slower and broader peaks, which explain the slower energy dissipation. This trend was not observed for sucrose and CA, where the sucrose‐0.80T, CA‐0.80T, and CA‐1.20T showed weaker network structure displaying varying energy dissipation from 0.1 to 1 Hz and 10 to 100 Hz in comparison with the control at concentrations below 2.60T. Therefore, this study highlights that the observed significant and variable differences in tan *δ* values due to the addition of sucrose, CA, and NaCl could potentially result in a different structural product type for the dysphagic patient, as cautioned earlier by Cho et al. (2015) and Payne et al. (2011).

### 3.2 Sensory Psychophysics

**FIGURE 3:**
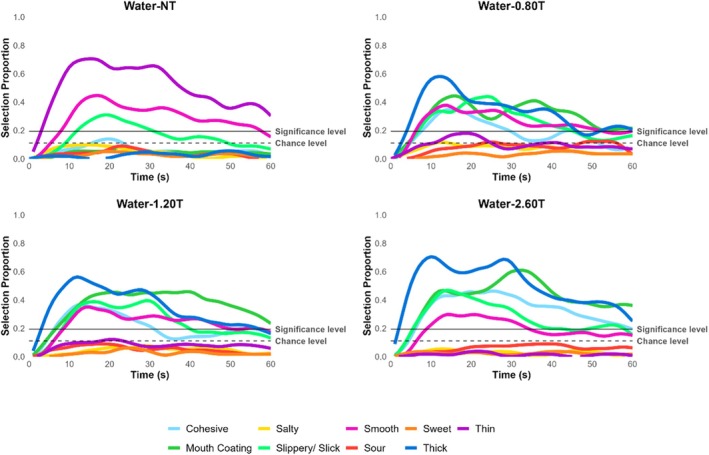
TCATA curves of water at varying thickener concentrations.

**FIGURE 4:**
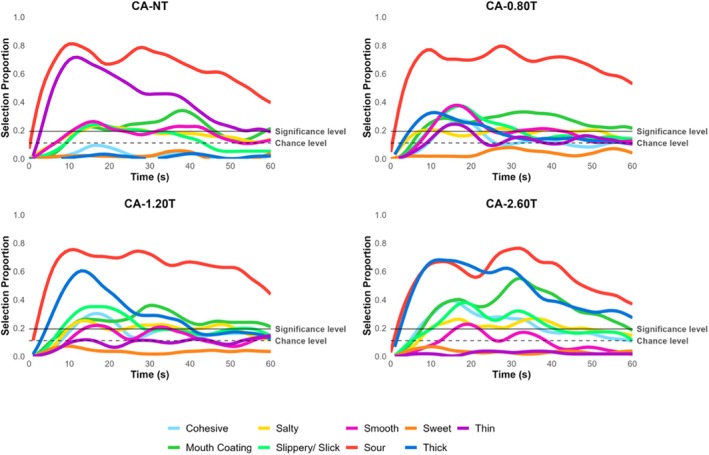
TCATA curves of NaCl solutions at varying thickener concentrations.

**FIGURE 5:**
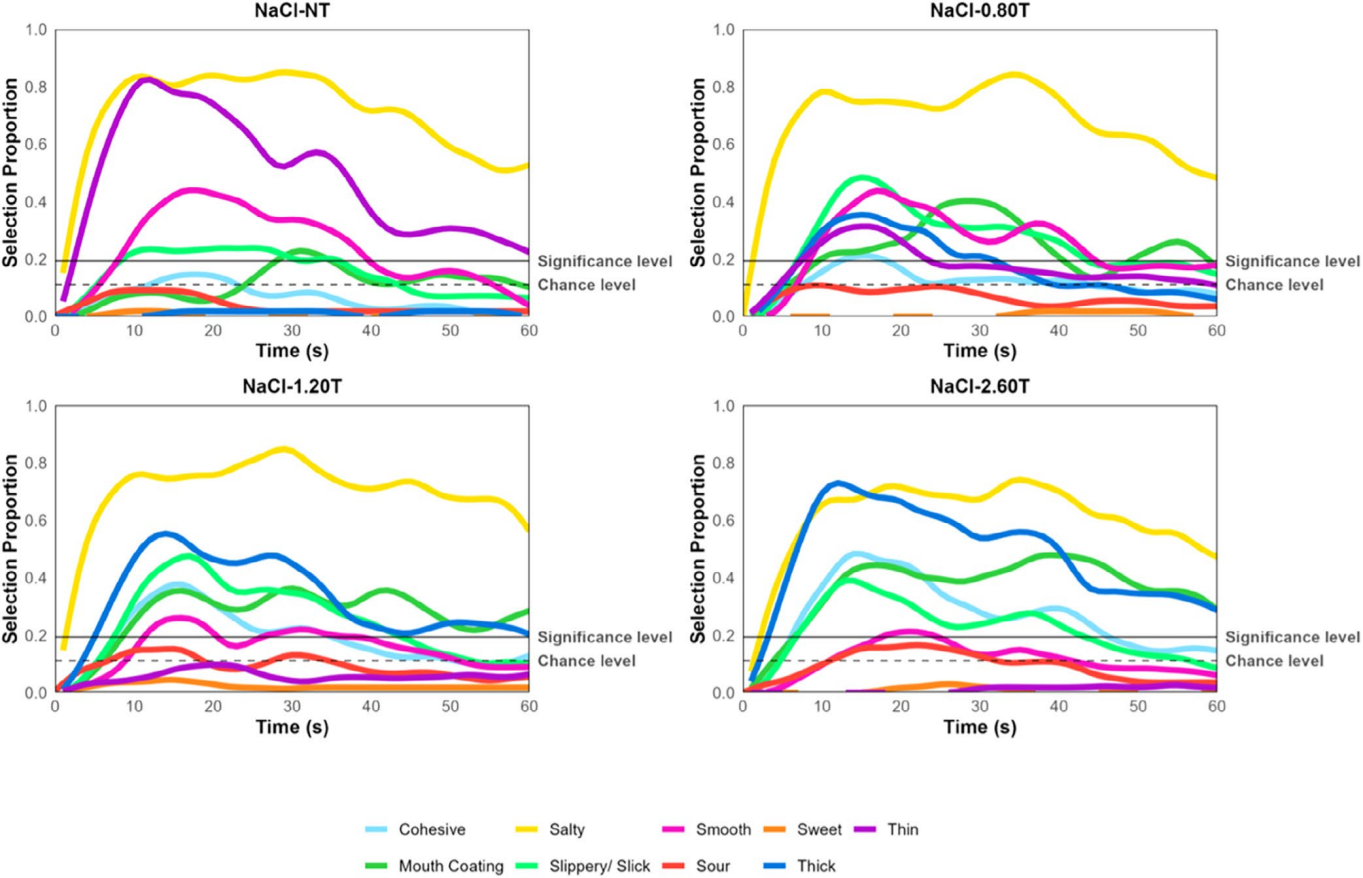
TCATA curves of CA solutions at varying thickener concentrations.

**FIGURE 6:**
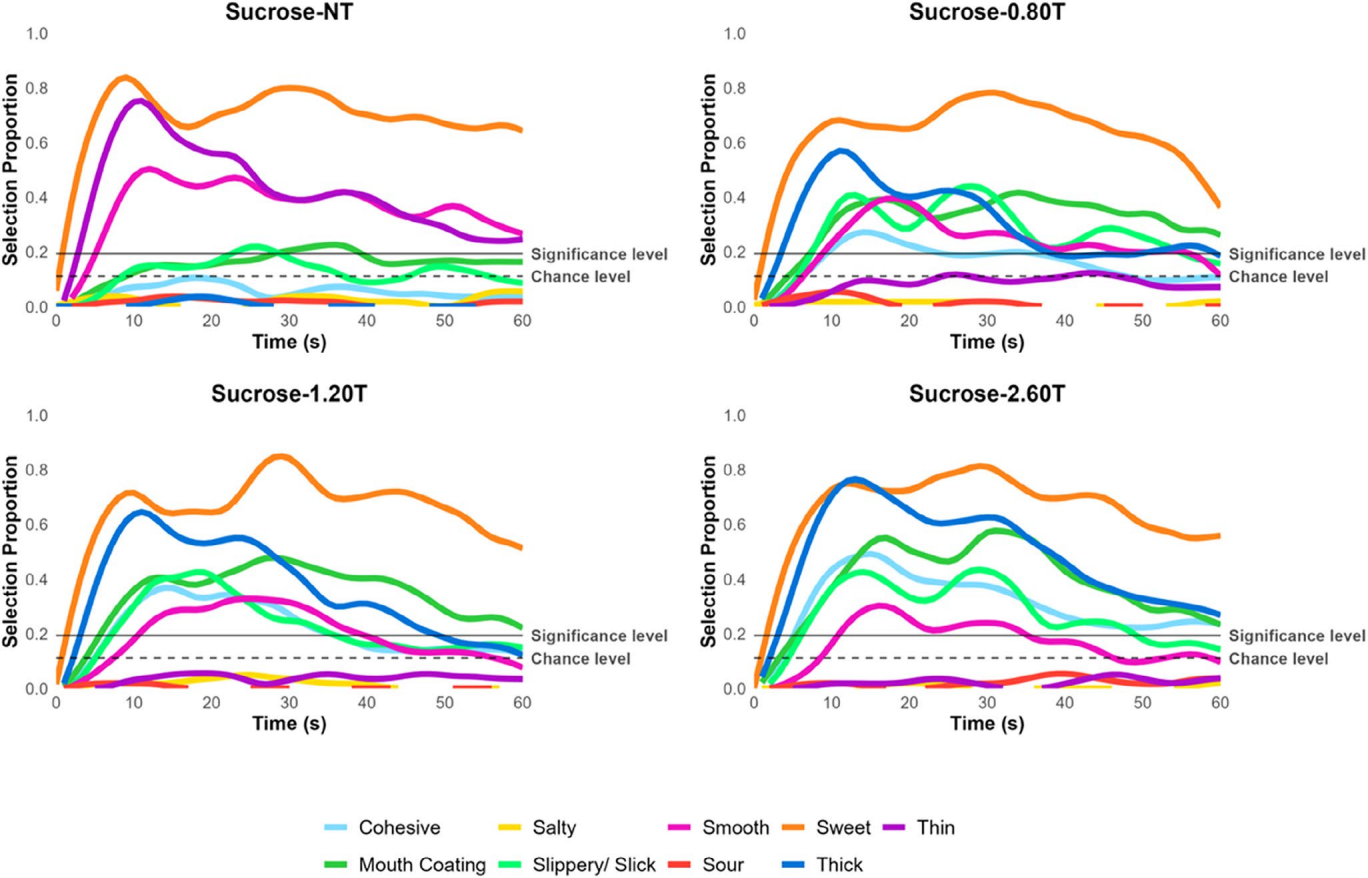
TCATA curves of sucrose solutions at varying thickener concentrations.

The temporal profile of attributes is reported in Figures 3, 4, 5, 6. TCATA parameters for taste attributes—maximum citation proportion (Cmax), area under the curve (AUC), citation end (Cend), time to max citation (Tmax), and T0.5 were extracted to explore their relationship (see Supporting Tables S4.1–4.5). Higher thickener concentrations resulted in a decrease in Cmax and AUC for the saltiness of NaCl samples. This effect was more pronounced for NaCl‐2.60T, which differed significantly from all other samples, including NaCl‐NT, NaCl‐0.80T, and NaCl‐1.20T (*p* < 0.05) (Figure 5). However, this trend was not consistently observable for the sourness of CA samples (Figure 4) or the sweetness of sucrose samples (Figure 6).

The possible explanations for the observed decrease in the AUC of the saltiness could be due to the NaCl lingering in the mouth for a longer duration (Green and Gelhard 1989) and the addition of a thickener restricting the NaCl molecules available for perception (Cook et al. 2002). Alternatively, the NaCl concentration was sufficient in the presence of a thickener for participants to notice a change in perceived saltiness. In contrast, the CA and sucrose, despite being higher without added thickener, became difficult to distinguish at higher thickener concentrations. This is supported by the detection or perception theory, suggesting that bulk rheological properties are more dominant in the mouth than taste attributes (Szczesniak 2002). However, the Cend results indicate that all NaCl samples were indistinguishable from each other at the end of the oral processing time, and T0.5 (referred to as lingering sensation) showed no difference in the time taken to decrease to half the citation proportion (see Supporting Table S4.1–4.5). Hence, current data fail to support the taste lingering mechanism.

Interestingly, Tmax revealed a delay in the time to reach the maximum citation proportion in the presence of a thickener for NaCl samples. However, this delay was more pronounced for CA and sucrose samples, suggesting that the NaCl concentration used here was perceived as relatively intense, and the increase in viscosity significantly altered dynamic perception. This finding is significant because the dynamic perception of sour and sweet tastes is more significantly affected than the perception of salt at the same thickener concentrations. While the oral processing time for liquids typically lasts 5–15 s (Lee III and Camps 1991; Zijlstra et al. 2008; De Wijk et al. 2008; Chen 2009), it may be challenging to experience the buildup of sour and sweet taste perception when thickened with a xanthan gum‐based thickener. If applied to clinical scenarios, such taste perception differences could influence the acceptance of thickened liquids.

Additionally, the end of sweetness for Sucrose‐2.60T varied significantly compared to Sucrose‐NT, indicating that adding a thickener reduced the duration of sweet taste perception at the end of the evaluation. All these combined results suggest that adding a thickener dynamically (in terms of duration, mouth linger, initial and aftertaste perception, continuity, etc.) alters the perception of salt, sour, and sweet tastes. Since the TCATA method does not directly measure intensity, perceived intensity ratings were reported on the generalized Visual Analog Scale (gVAS).

Black circles indicate mean (±SD) with gray circles indicating individual ratings. One‐way ANOVA and Tukey's HSD were performed for samples within the tastant solutions. Lowercase letters indicate intensity ratings significantly differed within the tastant solutions (*p* < 0.05).

**FIGURE 7:**
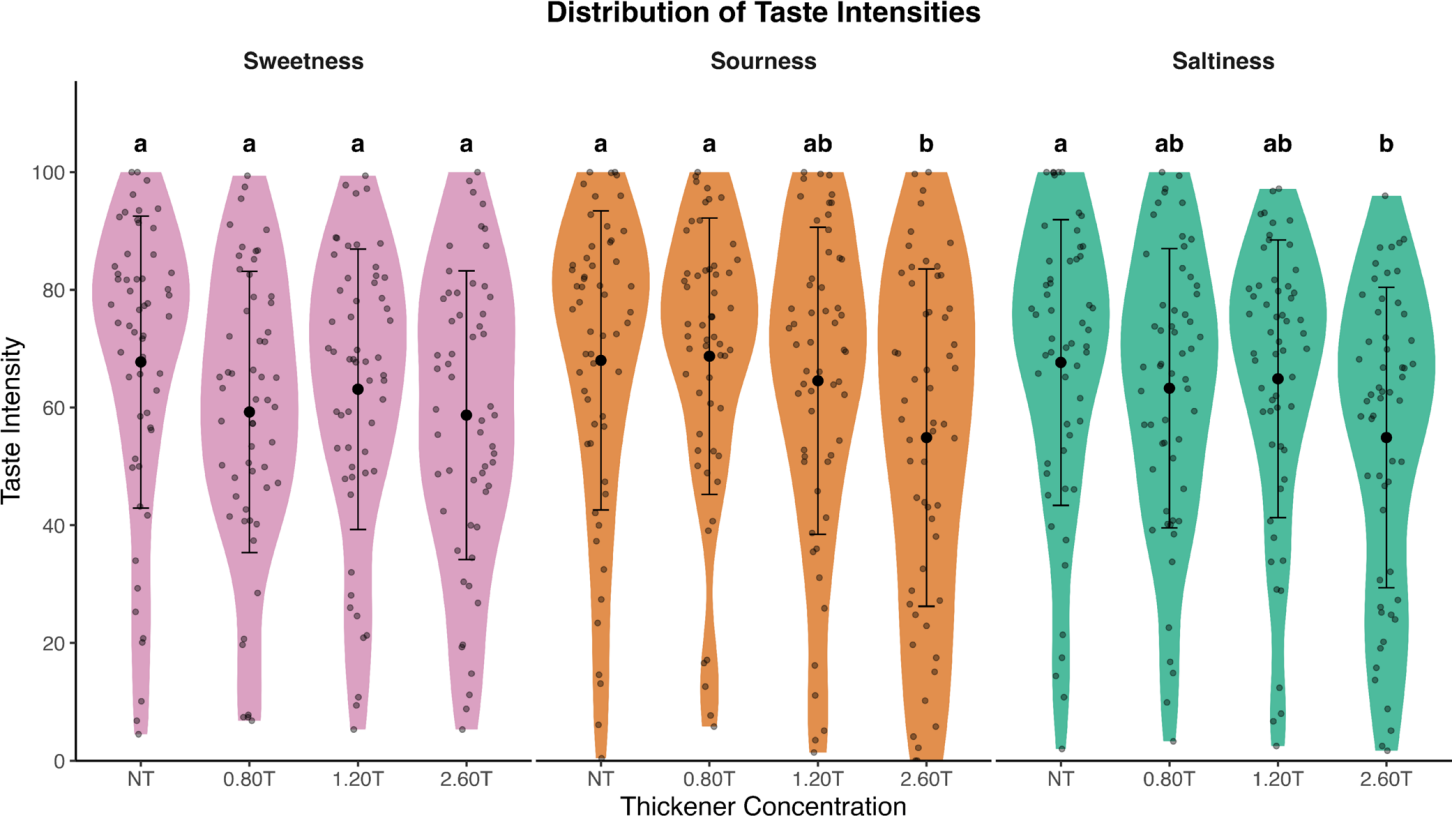
Distribution of taste intensity ratings across solutions.

Taste is a critical factor in determining the acceptance of any food product (Duffy and Bartoshuk 2000). Clinical studies (McCurtin et al. 2018; García‐Almeida et al. 2025) suggest that dysphagic food often presents with poor taste. Here, the distribution of taste intensity ratings is reported across tastant solutions at four thickener concentration levels, organized by tastant type (Figure 7). One‐way ANOVA revealed significant differences in sourness intensity for CA samples (*F*(3, 220) = 3.36, *p* = 0.0195) and salt intensity for NaCl samples (*F*(3, 220) = 2.87, *p* = 0.0375). However, the sweetness intensity for sucrose showed no significant differences across thickener levels (*F*(3, 220) = 1.66, *p* = 0.176).

Black circles refer to mean liking ratings, and gray circles refer to individual ratings. One‐way ANOVA and Tukey's HSD were performed for samples within the tastant solutions. Lowercase letters indicate intensity ratings significantly differed within the tastant solutions (*p* < 0.05).

**FIGURE 8:**
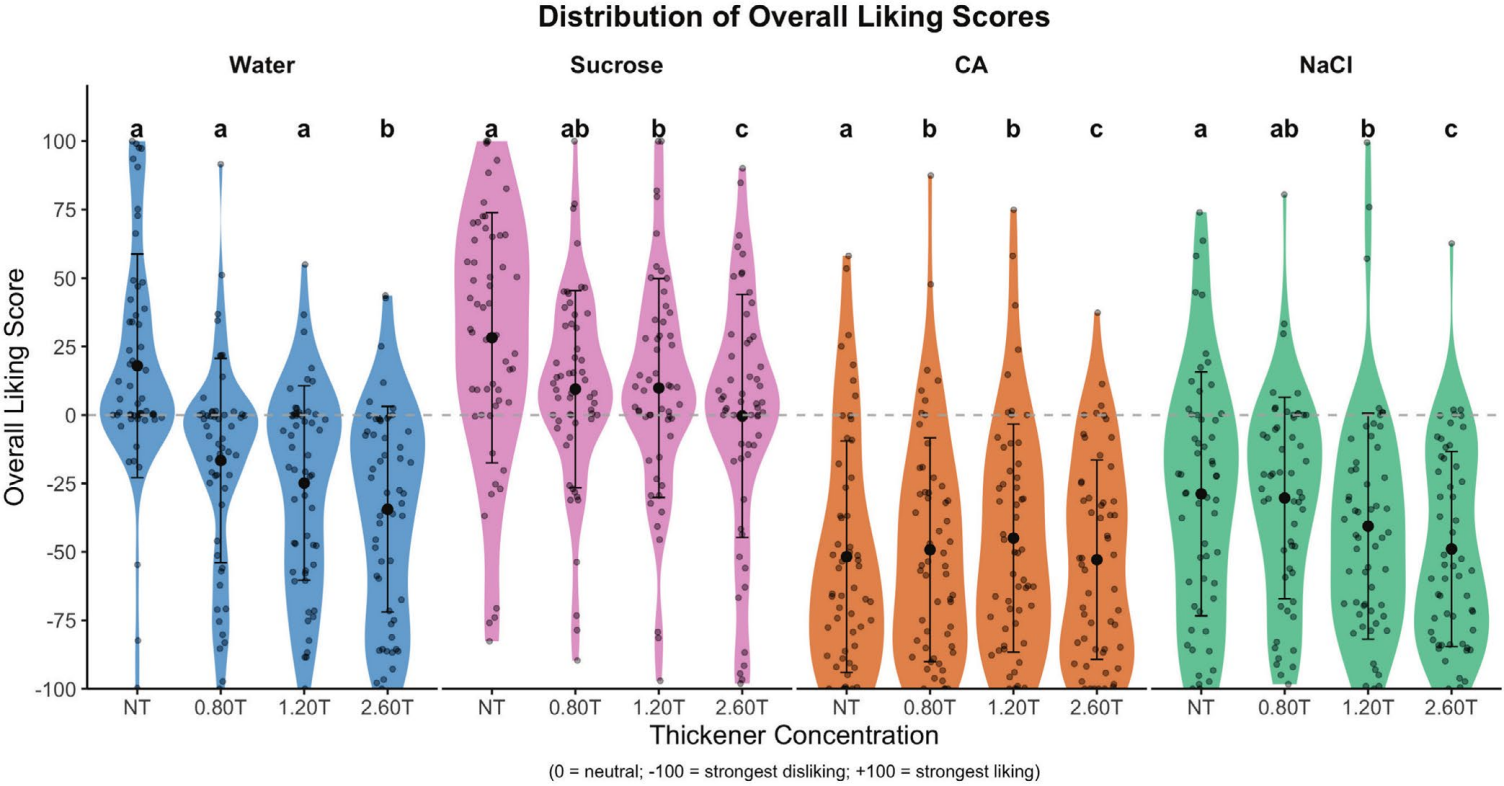
Distribution of overall liking ratings (±SD) for solutions.

**FIGURE 9:**
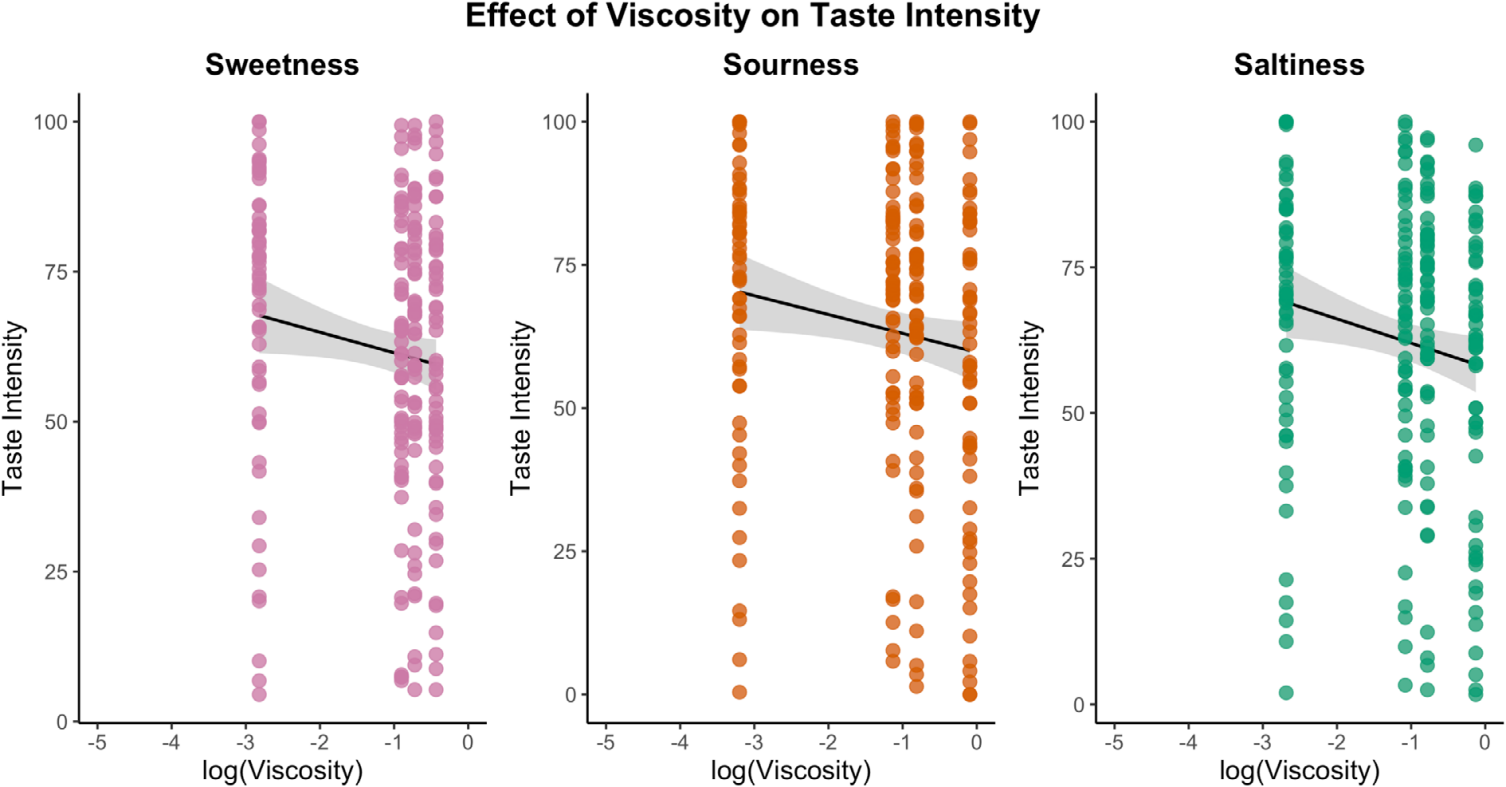
Effect of apparent viscosity on mean taste intensities. Regression lines indicate a progressive reduction in intensity with increasing viscosity.

Although the liking of model solutions differs from that of real food (Duffy and Bartoshuk 2000), it is important to explore how adding thickeners affects liking. The distribution of overall liking ratings for tastant solutions at four levels of thickener concentration is shown in Figure 8. A two‐way ANOVA revealed significant main effects for tastant (*F*(3, 880) = 102.8, *p* < 0.0001) and thickener concentration (*F*(3, 880) = 15.8, *p* < 0.0001), indicating that increases in thickener levels consistently reduced liking. Additionally, their interaction (*F*(9, 880) = 3.7, *p* = 0.001) suggests that the effect of the thickener varied across the tastant solutions. LMM further clarified these effects by examining the role of viscosity as a continuous predictor (discussed later, as shown in Figure 9).

Overall, the sensory results indicated that the tastant solutions significantly impacted the sensory properties when varying thickener levels. TCATA results showed no significant effect on the selection proportion of sour, salt, and sweet attributes with the addition of thickener. However, adding thickener effectively reduced the perception of salt and sourness intensity but did not affect sweetness perception, as indicated by ANOVA predictions using categorical thickener grouping.

### 3.3 Association Between Physical Measures and Sensory

LMM were conducted to investigate the relationship between intensity, liking, and instrumental textural measures. LMM revealed a significant effect of log‐transformed viscosity on taste intensity for each tastant (Supporting Table 5; Figure 9). An increase in viscosity corresponded with a reduction in sourness intensity *β* = −3.29, SE = 0.95, *t*(167) = −3.47, *p* < 0.001; saltiness *β* = −4.16, SE = 1.11, *t*(167) = −3.76, *p* < 0.001, and sweetness *β* = −3.43, SE = 0.86, *t*(167) = −3.99, *p* < 0.001. While saltiness showed the steepest decline, sweetness demonstrated the strongest overall model fit, with viscosity and panelist variability explaining 75.7% of variance compared to 61.7% for sourness and 60.3% for saltiness (Supporting Table 6).

While the viscosity effect alone was significant, it explained a slight variance (sweetness: 1.7%, saltiness: 2.5%, sourness: 2.1%), indicating that individual differences among panelists accounted for the most observable variability (Supporting Table 5). Model fit metrics reinforced these patterns, with sweetness showing the lowest Akaike Information Criterion (AIC = 1896.8), indicating superior model performance compared to saltiness (AIC = 1973.4) and sourness (AIC = 2000.0) (Supporting Table 6).

The gradual suppression of sweetness intensity is expected to reflect molecular competition between sucrose and xanthan gum for water‐binding sites, thereby reducing the free sucrose available for taste receptor interaction. In contrast, the consistent suppression in intensity for NaCl and CA is attributed to the charge screening effect and the strong interaction between the NaCl and CA taste solutions and the thickener. This aligns with previous work showing hydrocolloid‐specific taste modulation, particularly xanthan gum's stronger suppression of sourness and bitterness relative to sweetness (Pangborn et al. 1978). However, unlike hydroxypropyl methylcellulose (HPMC), which significantly reduces sweetness perception, as reported by Hollowood et al. (2002), xanthan gum's milder impact underscores the need for thickener‐specific sensory studies. In summary, adding a thickener significantly reduced the taste intensity (*p* < 0.001). However, individual differences dominated perception (conditional *R* ^2^ = 60%–76%), suggesting that molecular interactions (such as the interaction between tastants and thickener with water, hydrocolloid structure, etc.) rather than bulk viscosity drive changes in taste intensity. Therefore, additional parameters, such as spectroscopic studies, are needed to understand the effects of thickener addition on intensity.

**FIGURE 10:**
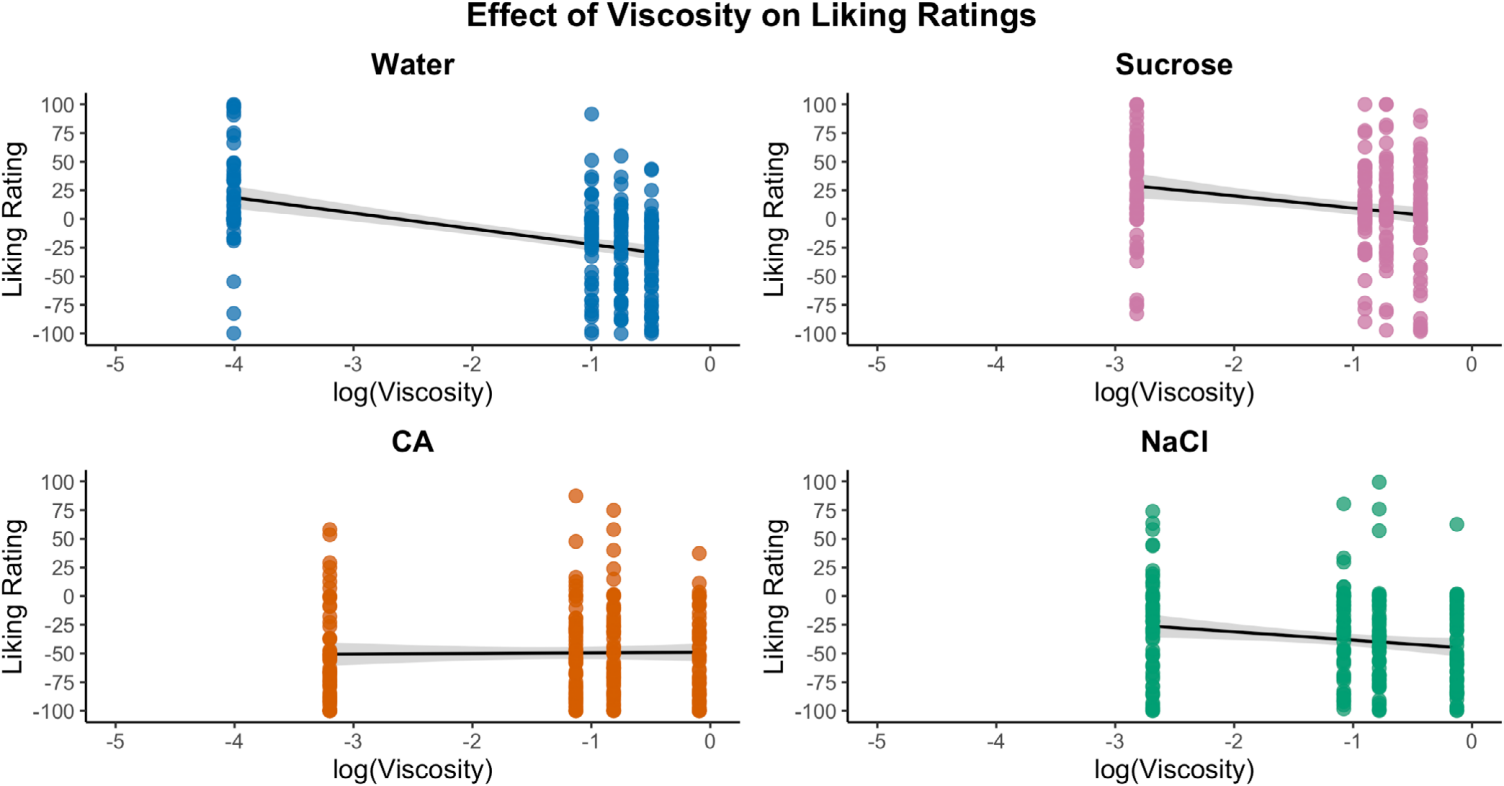
Effect of apparent viscosity on liking (±SD) across tastant solutions. Regression lines show progressive liking reductions with increasing viscosity.

Next, the LMM regression analysis performed between viscosity and liking indicated a significant reduction in overall liking (or disliking) (Figure 10). This analysis reveals a significant negative relationship between liking and viscosity for NaCl (AIC = 2178.8), sucrose (AIC = 2191.6), and CA (AIC = 2211.6). In contrast, water showed no significant effect due to a singular fit, as the model was unable to distinguish between panelist‐specific differences and residual variability. However, the magnitude and significance of these effects varied considerably across tastants (Figure 10, Supporting Table 7). For sucrose solutions, viscosity exerted a strong negative influence on liking (*β* = −10.6, SE = 1.82, *p* < 0.001), with the highest thickener concentration (2.60T) resulting in a reduction in liking by 25.4 compared to the no‐thickener control (NT) (*p* < 0.001) (Supporting Table 8). This relationship accounted for 64.5% of the variance in ratings, indicating that viscosity‐driven dislike significantly influenced participants' evaluations. Water, serving as a neutral control, exhibited the steepest viscosity‐dependent dislike trajectory (*β* = −47.8, SE = 5.00, *p* < 0.001), with viscosity alone explaining 49.4% of the variance, confirming that an increase in viscosity independently drives aversion in tasteless solutions. In contrast, saltiness (NaCl) and sourness (CA) had no significant effect on viscosity (*p* > 0.05). For NaCl, despite an 18.5 reduction in liking at higher thickener concentrations, i.e., 2.60T (*p* = 0.001), the overall viscosity slope was nonsignificant (*β* = −0.47, SE = 1.44, *p* = 0.74). Similarly, CA solutions showed minimal viscosity impact (*β* = −7.23, SE = 2.00, *p* = 0.40), with comparisons between NT revealing nonsignificant changes across thickener levels (*p* = 0.98).

Sucrose showed a gradual viscosity‐driven dislike, which coexists with stable sweetness perception, indicating the participants' aversiveness to viscous solutions. Furthermore, the results indicate that the additional thickener failed to significantly reduce liking, suggesting a ceiling effect (Duffy and Bartoshuk 2000), where the added thickener had no further influence on aversiveness. However, it is important to interpret these results with caution. Adding sucrose to a thickener may not necessarily improve acceptance, as a pure sweet solution is perceived differently from a complex beverage containing various tastes, textures, and flavors (Booth et al. 1987; Kim et al. 2014). Overall, LMM results reveal that viscosity significantly explains the overall liking scores (49.4% marginal *R* ^2^) for water and minimally for sucrose (marginal *R* ^2^ = 5.5%). In contrast, no significant viscosity effect was found for NaCl and CA (Supporting Table 7). Therefore, the conclusion that can be drawn here is that viscosity alone does not inform liking of tastant solutions due to taste‐texture complexations.

## 4 Limitations and Future Work

The design of this study aided in uncovering a new understanding of the interaction between the addition of ThickenUp clear thickener to achieve IDDSI levels (1–3) on the perception of sweetness of sucrose, sourness of CA, and saltiness of NaCl. Although this study provides new insights into the influence of thickener and physical texture properties on taste intensity and liking of simple tastant solutions, there is a need to explore more complex beverage matrices. While TCATA helped to identify changes in temporal perception, it did not capture dynamic changes in taste intensity. Future studies investigating dynamic changes in taste intensity would benefit from being paired with Time Intensity or Temporal Dominant Sensation methods, as studies building on this approach would help identify more effective strategies for improving preference without compromising the physical texture properties vital for safe swallowing. To better understand molecular dynamics, future research would benefit from utilizing a broader range of tastant concentrations, examining hydrocolloid‐specific interactions, and employing additional equipment (e.g., microscopic imaging, particle size measurements) to enhance the examination of interactions between tastants and thickeners.

## 5 Conclusion

This study aimed to quantify the impact of thickeners on simple model beverages to reflect three IDSSI thickener levels on the perception of sweet, sour, and salty tastes in healthy (nondysphagic) adults (*n* = 56). Expanding on prior work, these results demonstrate an interaction between texture and taste, integrating dynamic sensory profile and instrumental textural properties. Overall, the results demonstrate the complexity of interactions between different taste solutions and thickeners in shaping the final product characteristics. This is crucial for dysphagic research, as the behavior of the thickening medium can significantly alter both the rheology of the product and its sensory properties. The results of the present study underscore the need for a deeper understanding of the distinct interactions between thickeners and tastants, which can be crucial in developing products tailored for dysphagic patients. While it may not be feasible to customize thickener formulations for every beverage or food, it is achievable to identify dysphagic thickeners that do not significantly alter the rheological properties and preserve the taste and flavor characteristics of commonly prescribed or consumed beverages and foods.

## Author Contributions

Conceptualization: Alissa A. Nolden. Methodology: Gunalan Dhamodharan, Alissa A. Nolden, James Makame. Investigation, analysis: Gunalan Dhamodharan. Supervision: Alissa A. Nolden and James Makame. Writing – original draft: Gunalan Dhamodharan. Writing – reviewing and editing: Gunalan Dhamodharan, James Makame, and Alissa A. Nolden.

## Ethics Statement

This study was approved by the Institutional Review Board of The University of Massachusetts Amherst.

## Consent

Informed consent was obtained from all study participants.

## Conflicts of Interest

The authors declare no conflicts of interest.

## Supporting information


**Data S1:** Supplementary Tables.

## Data Availability

The data that support the findings of this study are available from the corresponding author upon reasonable request.
