# Supplementary material for: Texture‐Taste Interactions: Exploring the Effect of Thickener Concentration on the Sensory Perception of Sweet, Sour, and Salty Tastes
Source: J Texture Stud. 2025 Sep 21;56(5):e70042. doi: 10.1111/jtxs.70042 (PMC12450616; doi:10.1111/jtxs.70042)
Supplement: Supplementary file 1 — Data S1: Supplementary Tables. [file JTXS-56-e70042-s001.docx]

SUPPLEMENTARY MATERIALS:

Supplementary Table: S1. Linear mixed model results were obtained for the TCATA parameter AUC (Area Under Curve) and COF. Low marginal R^2^ indicates that tribology (COF) did not inform temporal measures in this study.

| Attribute | Estimate | SE | t-value | p-value | AIC | R² Conditional | R² Marginal | Panelist Variance | Sample Variance |
| --- | --- | --- | --- | --- | --- | --- | --- | --- | --- |
| Cohesive | -0.357 | 0.90 | -0.395 | 0.693 | 96303 | 0.680 | 0.000027 | 48.0 | 0.893 |
| Slippery | -0.413 | 1.49 | -0.276 | 0.783 | 113733 | 0.599 | 0.000015 | 101.0 | 0.962 |
| Thin | -0.303 | 1.86 | -0.163 | 0.870 | 118957 | 0.593 | 0.000006 | 130.0 | 7.39 |
| Mouth Coating | 0.226 | 1.81 | 0.125 | 0.900 | 117874 | 0.507 | 0.000004 | 77.2 | 14.0 |
| Smooth | -0.197 | 1.93 | -0.102 | 0.919 | 120024 | 0.610 | 0.000002 | 135.0 | 23.2 |
| Sour | 0.063 | 1.74 | 0.036 | 0.971 | 116457 | 0.823 | 0.0000001 | 36.2 | 341.0 |
| Thick | 0.019 | 0.60 | 0.033 | 0.974 | 83556 | 0.398 | 0.0000004 | 6.64 | 0.279 |
| Salty | -0.029 | 1.90 | -0.015 | 0.987 | 119198 | 0.810 | 0.00000003 | 39.6 | 371.0 |
| Sweet | -0.007 | 1.34 | -0.005 | 0.995 | 108019 | 0.900 | 0.000000002 | 16.4 | 416.0 |

Supplementary Table: S2. Linear mixed model results were performed between overall liking responses and COF. Low marginal R^2^ indicates that tribology (COF) did not inform overall liking in this study.

| Sample | Estimate | SE | t-value | p-value | AIC | R² Conditional | R² Marginal | Panelist Variance |
| --- | --- | --- | --- | --- | --- | --- | --- | --- |
| NaCl-2.60T | −3.14×10^−10^ | 5.29×10^−6^ | −0.0000594 | 1.000 | -81278 | 1.000 | 2.32×10^−24^ | 1.00 |
| Sucrose-0.80T | 9.76×10^−11^ | 2.38×10^−6^ | 0.0000410 | 1.000 | -86220 | 1.000 | 3.81×10^−25^ | 1.00 |
| NaCl-NT | −1.25×10^−10^ | 3.55×10^−6^ | −0.0000352 | 1.000 | -81254 | 1.000 | 5.57×10^−25^ | 1.00 |
| CA-2.60T | −2.83×10^−11^ | 1.10×10^−6^ | −0.0000258 | 1.000 | -82854 | 1.000 | 3.53×10^−25^ | 1.00 |
| NaCl-1.20T | 1.18×10^−10^ | 5.04×10^−6^ | 0.0000235 | 1.000 | -81332 | 1.000 | 3.03×10^−25^ | 1.00 |
| Sucrose-NT | −2.82×10^−11^ | 2.32×10^−6^ | −0.0000122 | 1.000 | -83222 | 1.000 | 4.84×10^−26^ | 1.00 |
| Sucrose-2.60T | 1.11×10^−11^ | 1.22×10^−6^ | 0.00000908 | 1.000 | -87543 | 1.000 | 1.02×10^−26^ | 1.00 |
| Water-0.80T | 4.42×10^−12^ | 1.06×10^−6^ | 0.00000419 | 1.000 | -87377 | 1.000 | 3.23×10^−27^ | 1.00 |
| Water-2.60T | −2.90×10^−11^ | 4.63×10^−5^ | −0.000000627 | 1.000 | -57929 | 1.000 | 1.20×10^−25^ | 1.00 |
| CA-0.80T | −1.47×10^−11^ | 2.55×10^−5^ | −0.000000579 | 1.000 | -56722 | 1.000 | 1.17×10^−25^ | 1.00 |
| CA-NT | 5.33×10^−11^ | 1.12×10^−4^ | 0.000000478 | 1.000 | -56573 | 1.000 | 7.76×10^−26^ | 1.00 |
| Water-NT | −1.93×10^−11^ | 5.13×10^−5^ | −0.000000377 | 1.000 | -57023 | 1.000 | 4.62×10^−26^ | 1.00 |
| NaCl-0.80T | 1.74×10^−11^ | 7.05×10^−6^ | 0.000000247 | 1.000 | -57948 | 1.000 | 1.94×10^−26^ | 1.00 |
| Water-1.20T | −6.17×10^−12^ | 4.53×10^−5^ | −0.000000136 | 1.000 | -58492 | 1.000 | 5.50×10^−27^ | 1.00 |
| Sucrose-1.20T | −5.45×10^−12^ | 6.72×10^−5^ | −0.000000081 | 1.000 | -58240 | 1.000 | 1.64×10^−27^ | 1.00 |
| CA-1.20T | 2.35×10^−13^ | 2.05×10^−5^ | 0.0000000115 | 1.000 | -59022 | 1.000 | 2.46×10^−29^ | 1.00 |

Supplementary Table: S3. Linear mixed model results were performed between taste intensity responses and COF. Low marginal R^2^ indicates that tribology (COF) did not inform taste intensity in this study.

| Sample | Estimate | SE | t-value | p-value | AIC | R² Conditional | R² Marginal | Panelist Variance |
| --- | --- | --- | --- | --- | --- | --- | --- | --- |
| CA-NT | -2.27e^-10^ | 11.9 | -1.92e^-11^ | 1.000 | 120000 | 0.121 | 2.67e^-26^ | 0.121 |
| NaCl-0.80T | 1.44e^-10^ | 8.07 | 1.79e^-11^ | 1.000 | 117000 | 0.128 | 2.30e^-26^ | 0.128 |
| Sucrose-0.80T | -1.29e^-10^ | 8.98 | -1.44e^-11^ | 1.000 | 116000 | 0.101 | 1.53e^-26^ | 0.101 |
| NaCl-1.20T | -1.57e^-10^ | 11.0 | -1.42e-^11^ | 1.000 | 117000 | 0.187 | 1.36e^-26^ | 0.187 |
| CA-0.80T | -3.74e^-11^ | 2.70 | -1.38e^-11^ | 1.000 | 119000 | 0.140 | 1.36e^-26^ | 0.140 |
| CA-1.20T | -3.18e^-11^ | 2.77 | -1.15e^-11^ | 1.000 | 118000 | 0.156 | 9.20e^-27^ | 0.156 |
| NaCl-2.60T | 1.15e^-10^ | 10.5 | 1.09e^-11^ | 1.000 | 115000 | 0.117 | 8.67e^-27^ | 0.117 |
| Water-2.60T | -9.52e^-12^ | 0.909 | -1.05e^-11^ | 1.000 | 74800 | 0.727 | 2.48e^-27^ | 0.727 |
| NaCl-NT | -8.68e^-11^ | 8.31 | -1.04e^-11^ | 1.000 | 119000 | 0.0769 | 8.33e^-27^ | 0.0769 |
| Sucrose-NT | -5.37e^-11^ | 6.95 | -7.72e^-12^ | 1.000 | 119000 | 0.0715 | 4.57e^-27^ | 0.0715 |
| Water-NT | -8.84e^-12^ | 1.22 | -7.22e^-12^ | 1.000 | 82200 | 0.418 | 2.51e^-27^ | 0.418 |
| Sucrose-1.20T | -3.28e^-11^ | 5.50 | -5.96e^-12^ | 1.000 | 116000 | 0.129 | 2.55e^-27^ | 0.129 |
| Sucrose-2.60T | 4.28e^-11^ | 8.12 | 5.27e^-12^ | 1.000 | 118000 | 0.0748 | 2.13e^-27^ | 0.0748 |
| Water-0.80T | -2.47e^-12^ | 1.49 | -1.66e^-12^ | 1.000 | 88500 | 0.489 | 1.16e^-28^ | 0.489 |
| Water-1.20T | 7.51e^-13^ | 1.22 | 6.16e^-13^ | 1.000 | 80700 | 0.693 | 9.62e^-30^ | 0.693 |
| CA-2.60T | 1.30e^-13^ | 2.67 | 4.88e^-14^ | 1.000 | 115000 | 0.178 | 1.65e^-31^ | 0.178 |

Supplementary Table S4.1. Summary of a TCATA parameter- Cmax (prolongation of the taste or mouthfeel perception) for different tastant solutions varying in viscosities (i.e., thickener concentrations). The lowercase letters indicate significant differences between the samples in the same tastant solution group (p<0.05).

| Attribute | Water (Control) | | | | CA | | | | NaCl | | | | Sucrose | | | |
| --- | --- | --- | --- | --- | --- | --- | --- | --- | --- | --- | --- | --- | --- | --- | --- | --- |
|  | Water-NT | Water 0.80T | Water 1.20T | Water 2.60T | CA-NT | CA 0.80T | CA 1.20T | CA 2.60T | NaCl-NT | NaCl 0.80T | NaCl 1.20T | NaCl 2.60T | Sucrose-NT | Sucrose 0.80T | Sucrose 1.20T | Sucrose 2.60T |
| Cohesive | 0.143ᶜᵈ | 0.357ᵇ | 0.393ᵇ | 0.482ᵃ | 0.107ᶜᵈ | 0.268ᵇᶜ | 0.304ᵇ | 0.429ᵃ | 0.143ᶜᵈ | 0.214ᶜᵈ | 0.411ᵇ | 0.536ᵃ | 0.109ᶜᵈ | 0.286ᵇᶜ | 0.393ᵇ | 0.518ᵃ |
| Mouth Coating | 0.089ᵈ | 0.464ᵇ | 0.482ᵇ | 0.625ᵃ | 0.357ᵇᶜ | 0.339ᵇᶜ | 0.375ᵇᶜ | 0.589ᵃ | 0.250ᵇᶜ | 0.411ᵇ | 0.375ᵇ | 0.500ᵃ | 0.236ᶜᵈ | 0.446ᵇ | 0.500ᵇ | 0.607ᵃ |
| Salty | 0.107ᵇ | 0.125ᵃ | 0.089ᵇ | 0.054ᵇᶜ | 0.250ᵇ | 0.232ᵇ | 0.250ᵇ | 0.304ᵃ | 0.875ᵃ | 0.857ᵃ | 0.875ᵃ | 0.786ᵇ | 0.055ᵃ | 0.018ᶜᵈ | 0.054ᵃ | 0.018ᶜᵈ |
| Slippery | 0.321ᵇᶜ | 0.464ᵃ | 0.429ᵇ | 0.482ᵃ | 0.268ᵇᶜ | 0.393ᵃ | 0.375ᵃ | 0.393ᵃ | 0.250ᵇᶜ | 0.482ᵃ | 0.500ᵃ | 0.411ᵇ | 0.236ᵇᶜ | 0.446ᵃ | 0.446ᵃ | 0.446ᵃ |
| Smooth | 0.464ᵃ | 0.429ᵃ | 0.375ᵇ | 0.321ᵇᶜ | 0.268ᵇ | 0.375ᵃ | 0.214ᵇᶜ | 0.250ᵇᶜ | 0.464ᵃ | 0.464ᵃ | 0.268ᵇᶜ | 0.214ᵇᶜ | 0.527ᵃ | 0.393ᵇ | 0.357ᵇᶜ | 0.304ᵇᶜ |
| Sour | 0.107ᵇ | 0.143ᵃ | 0.089ᵇᶜ | 0.089ᵇᶜ | 0.821ᵃ | 0.821ᵃ | 0.768ᵃ | 0.804ᵃ | 0.089ᵇᶜ | 0.107ᵇᶜ | 0.161ᵃ | 0.179ᵃ | 0.036ᵇᶜ | 0.054ᵃ | 0.018ᶜᵈ | 0.054ᵃ |
| Sweet | 0.054ᵇ | 0.054ᵇ | 0.071ᵃ | 0.036ᵇᶜ | 0.054ᵇᶜ | 0.089ᵃ | 0.071ᵇ | 0.071ᵇ | 0.018ᶜᵈ | 0.018ᶜᵈ | 0.054ᵃ | 0.036ᵇᶜ | 0.855ᵃ | 0.804ᵃ | 0.857ᵃ | 0.839ᵃ |
| Thick | 0.054ᵈ | 0.589ᵇ | 0.607ᵇ | 0.732ᵃ | 0.036ᵈ | 0.339ᵇᶜ | 0.625ᵇ | 0.696ᵃ | 0.018ᵈ | 0.357ᵇᶜ | 0.554ᵇ | 0.750ᵃ | 0.036ᵈ | 0.589ᵇ | 0.679ᵇ | 0.804ᵃ |
| Thin | 0.714ᵃ | 0.179ᶜᵈ | 0.125ᵈ | 0.036ᵈ | 0.732ᵃ | 0.250ᶜᵈ | 0.143ᵈ | 0.036ᵈ | 0.875ᵃ | 0.321ᶜᵈ | 0.107ᵈ | 0.036ᵈ | 0.764ᵃ | 0.125ᵈ | 0.054ᵈ | 0.054ᵈ |

Supplementary Table S4.2. Summary of a TCATA parameter-AUC (area under the curve) for different tastant solutions varying thickener concentrations. The lowercase letters indicate significant differences between the samples in the same tastant solution group (p<0.05).

| Attribute | Water (Control) | | | | CA | | | | NaCl | | | | Sucrose | | | |
| --- | --- | --- | --- | --- | --- | --- | --- | --- | --- | --- | --- | --- | --- | --- | --- | --- |
|  | Water-NT | Water 0.80T | Water 1.20T | Water 2.60T | CA-NT | CA 0.80T | CA 1.20T | CA 2.60T | NaCl-NT | NaCl 0.80T | NaCl 1.20T | NaCl 2.60T | Sucrose-NT | Sucrose 0.80T | Sucrose 1.20T | Sucrose 2.60T |
| Cohesive | 3.59ᵈ | 10.91ᵇᶜ | 12.58ᵇᶜ | 19.67ᵃ | 1.61ᵈ | 7.49ᵇᶜ | 9.85ᵇ | 12.86ᵃ | 3.77ᶜᵈ | 7.04ᵇᶜ | 11.17ᵇᶜ | 16.05ᵃ | 3.29ᵈ | 9.63ᵇᶜ | 12.83ᵇᶜ | 18.38ᵃ |
| Mouth Coating | 2.73ᵈ | 17.86ᵇ | 21.54ᵇ | 24.54ᵃ | 11.55ᵇᶜ | 14.88ᵇ | 14.19ᵇ | 19.96ᵃ | 6.77ᶜᵈ | 13.99ᵇᶜ | 16.16ᵇ | 21.88ᵃ | 9.23ᶜᵈ | 19.01ᵇ | 20.52ᵇ | 23.40ᵃ |
| Salty | 2.71ᵇᶜ | 4.13ᵃ | 2.63ᵇᶜ | 1.63ᶜᵈ | 9.86ᵇ | 10.68ᵃ | 10.76ᵃ | 11.55ᵃ | 42.30ᵃ | 40.67ᵃ | 42.07ᵃ | 36.44ᵇ | 1.30ᵃ | 0.69ᵇᶜ | 1.04ᵇ | 0.24ᵈ |
| Slippery | 9.43ᵇᶜ | 15.08ᵃ | 14.44ᵇ | 16.32ᵃ | 7.87ᵇᶜ | 11.38ᵇ | 12.38ᵃ | 13.13ᵃ | 9.16ᵇᶜ | 15.70ᵃ | 14.92ᵃ | 13.03ᵇ | 7.54ᵇᶜ | 16.13ᵃ | 13.39ᵇ | 16.29ᵃ |
| Smooth | 17.04ᵃ | 14.83ᵇ | 13.94ᵇ | 11.50ᵇᶜ | 10.24ᵃ | 11.15ᵃ | 7.74ᵇᶜ | 5.87ᵇᶜ | 14.25ᵃ | 14.88ᵃ | 9.17ᵇᶜ | 6.83ᵇᶜ | 21.85ᵃ | 13.68ᵇᶜ | 11.70ᵇᶜ | 9.88ᵇᶜ |
| Sour | 2.13ᵇᶜ | 4.86ᵃ | 3.01ᵇᶜ | 3.47ᵇ | 38.62ᵃ | 40.22ᵃ | 37.93ᵃ | 34.29ᵇ | 2.24ᵇᶜ | 3.98ᵇ | 5.49ᵃ | 5.52ᵃ | 0.92ᵇ | 0.79ᵇᶜ | 0.21ᵈ | 1.23ᵃ |
| Sweet | 1.63ᵃ | 1.80ᵃ | 1.51ᵇ | 1.40ᵇ | 1.09ᵇᶜ | 2.36ᵃ | 2.20ᵃ | 1.96ᵇ | 0.18ᵈ | 0.36ᶜᵈ | 1.31ᵃ | 0.36ᶜᵈ | 41.75ᵃ | 37.96ᵃ | 39.27ᵃ | 39.54ᵃ |
| Thick | 1.58ᵈ | 19.69ᵇᶜ | 20.79ᵇᶜ | 29.88ᵃ | 0.83ᵈ | 10.86ᶜᵈ | 17.36ᵇᶜ | 28.19ᵃ | 0.66ᵈ | 10.51ᶜᵈ | 19.71ᵇᶜ | 29.01ᵃ | 0.36ᵈ | 18.41ᵇᶜ | 21.82ᵇ | 29.33ᵃ |
| Thin | 30.18ᵃ | 6.02ᵈ | 4.79ᵈ | 0.89ᵈ | 25.07ᵃ | 7.84ᶜᵈ | 5.64ᶜᵈ | 1.29ᵈ | 29.23ᵃ | 10.43ᶜᵈ | 3.34ᵈ | 0.62ᵈ | 25.29ᵃ | 4.89ᵈ | 2.18ᵈ | 1.21ᵈ |

Supplementary Table 4.3. Summary of a TCATA parameter: Cend (citation proportion at the end of the TCATA curve) for different tastant solutions varying thickener concentrations. The lowercase letters indicate significant differences between the samples in the same tastant solution group (p<0.05).

| Attribute | Water (Control) | | | | CA | | | | NaCl | | | | Sucrose | | | |
| --- | --- | --- | --- | --- | --- | --- | --- | --- | --- | --- | --- | --- | --- | --- | --- | --- |
|  | Water-NT | Water 0.80T | Water 1.20T | Water 2.60T | CA-NT | CA 0.80T | CA 1.20T | CA 2.60T | NaCl-NT | NaCl 0.80T | NaCl 1.20T | NaCl 2.60T | Sucrose-NT | Sucrose 0.80T | Sucrose 1.20T | Sucrose 2.60T |
| Cohesive | 0.00ᵈ | 0.07ᶜᵈ | 0.16ᵇ | 0.20ᵃ | 0.00ᵈ | 0.13ᵃ | 0.13ᵃ | 0.11ᵇ | 0.00ᵈ | 0.07ᵇᶜ | 0.13ᵇ | 0.14ᵃ | 0.04ᵈ | 0.11ᵇᶜ | 0.13ᵇᶜ | 0.23ᵃ |
| Mouth Coating | 0.04ᵈ | 0.21ᵇᶜ | 0.23ᵇᶜ | 0.36ᵃ | 0.21ᵃ | 0.20ᵃ | 0.20ᵃ | 0.18ᵇ | 0.11ᶜᵈ | 0.16ᵇᶜ | 0.29ᵃ | 0.27ᵃ | 0.16ᵇ | 0.23ᵃ | 0.21ᵃ | 0.23ᵃ |
| Salty | 0.00ᵈ | 0.04ᵃ | 0.02ᵇᶜ | 0.00ᵈ | 0.14ᵃ | 0.11ᵇ | 0.13ᵇ | 0.14ᵃ | 0.54ᵃ | 0.48ᵃ | 0.54ᵃ | 0.48ᵃ | 0.05ᵃ | 0.02ᶜᵈ | 0.00ᵈ | 0.02ᶜᵈ |
| Slippery/Slick | 0.07ᵇᶜ | 0.16ᵃ | 0.13ᵇ | 0.14ᵇ | 0.05ᶜᵈ | 0.14ᵃ | 0.14ᵃ | 0.11ᵇ | 0.07ᵇᶜ | 0.14ᵃ | 0.09ᵇᶜ | 0.09ᵇᶜ | 0.09ᵇᶜ | 0.16ᵃ | 0.14ᵇ | 0.14ᵇ |
| Smooth | 0.16ᵇ | 0.20ᵃ | 0.16ᵇ | 0.14ᵇ | 0.13ᵃ | 0.13ᵃ | 0.13ᵃ | 0.02ᵈ | 0.04ᵈ | 0.18ᵃ | 0.09ᵇᶜ | 0.05ᶜᵈ | 0.27ᵃ | 0.11ᶜᵈ | 0.07ᶜᵈ | 0.09ᶜᵈ |
| Sour | 0.00ᵈ | 0.04ᵇᶜ | 0.02ᶜᵈ | 0.05ᵃ | 0.41ᵇ | 0.52ᵃ | 0.43ᵇ | 0.39ᵇ | 0.02ᶜᵈ | 0.04ᵇᶜ | 0.05ᵃ | 0.04ᵇᶜ | 0.02ᵇᶜ | 0.00ᵈ | 0.00ᵈ | 0.04ᵃ |
| Sweet | 0.02ᵇᶜ | 0.04ᵃ | 0.02ᵇᶜ | 0.02ᵇᶜ | 0.04ᵃ | 0.04ᵃ | 0.04ᵃ | 0.04ᵃ | 0.00ᵈ | 0.00ᵈ | 0.02ᵃ | 0.00ᵈ | 0.62ᵃ | 0.36ᵇᶜ | 0.50ᵇ | 0.55ᵇ |
| Thick | 0.02ᵈ | 0.20ᵇ | 0.16ᵇᶜ | 0.25ᵃ | 0.02ᵈ | 0.11ᶜᵈ | 0.11ᶜᵈ | 0.27ᵃ | 0.00ᵈ | 0.05ᵈ | 0.20ᵇ | 0.27ᵃ | 0.00ᵈ | 0.18ᵇᶜ | 0.11ᶜᵈ | 0.27ᵃ |
| Thin | 0.29ᵃ | 0.07ᶜᵈ | 0.05ᵈ | 0.00ᵈ | 0.18ᵃ | 0.11ᵇᶜ | 0.14ᵇ | 0.02ᵈ | 0.21ᵃ | 0.11ᵇᶜ | 0.07ᶜᵈ | 0.02ᵈ | 0.25ᵃ | 0.07ᶜᵈ | 0.04ᵈ | 0.04ᵈ |

Supplementary Table 4.4. Summary of a TCATA parameter: Tmax (buildup of sensation) for different tastant solutions varying thickener concentrations. The lowercase letters indicate significant differences between the samples in the same tastant solution group (p<0.05).

| Attribute | Water (Control) | | | | CA | | | | NaCl | | | | Sucrose | | | |
| --- | --- | --- | --- | --- | --- | --- | --- | --- | --- | --- | --- | --- | --- | --- | --- | --- |
|  | Water-NT | Water 0.80T | Water 1.20T | Water 2.60T | CA-NT | CA 0.80T | CA 1.20T | CA 2.60T | NaCl-NT | NaCl 0.80T | NaCl 1.20T | NaCl 2.60T | Sucrose-NT | Sucrose 0.80T | Sucrose 1.20T | Sucrose 2.60T |
| Cohesive | 19 | 13 | 12 | 19 | 15 | 16 | 15 | 15 | 14 | 12 | 16 | 14 | 16 | 13 | 15 | 16 |
| Mouth Coating | 22 | 16 | 22 | 29 | 38 | 29 | 29 | 32 | 31 | 25 | 30 | 38 | 36 | 35 | 27 | 30 |
| Salty | 12 | 12 | 28 | 10 | 17 | 39 | 12 | 17 | 28 | 34 | 29 | 35 | 54 | 1 | 23 | 16 |
| Slippery/Slick | 19 | 25 | 30 | 13 | 18 | 18 | 19 | 17 | 13 | 14 | 17 | 12 | 25 | 26 | 19 | 14 |
| Smooth | 17 | 14 | 14 | 14 | 15 | 15 | 14 | 18 | 16 | 17 | 18 | 18 | 13 | 16 | 22 | 15 |
| Sour | 23 | 49 | 12 | 26 | 9 | 10 | 10 | 33 | 6 | 7 | 10 | 16 | 12 | 8 | 3 | 37 |
| Sweet | 17 | 18 | 23 | 5 | 33 | 30 | 7 | 9 | 7 | 35 | 14 | 25 | 8 | 31 | 29 | 30 |
| Thick | 28 | 14 | 11 | 10 | 17 | 11 | 13 | 11 | 14 | 15 | 13 | 12 | 16 | 11 | 12 | 13 |
| Thin | 14 | 15 | 19 | 17 | 12 | 14 | 57 | 20 | 11 | 14 | 20 | 46 | 10 | 24 | 14 | 45 |

Supplementary Table 4.5. Summary of a TCATA parameter: T0.5 (lingering of sensation) for different tastant solutions varying in thickener concentrations. The lowercase letters indicate significant differences between the samples in the same row (p<0.05).

| Attribute | Water (Control) | | | | CA | | | | NaCl | | | | Sucrose | | | |
| --- | --- | --- | --- | --- | --- | --- | --- | --- | --- | --- | --- | --- | --- | --- | --- | --- |
|  | Water-NT | Water 0.80T | Water 1.20T | Water 2.60T | CA-NT | CA 0.80T | CA 1.20T | CA 2.60T | NaCl-NT | NaCl 0.80T | NaCl 1.20T | NaCl 2.60T | Sucrose-NT | Sucrose 0.80T | Sucrose 1.20T | Sucrose 2.60T |
| Cohesive | 9 | 7 | 6 | 7 | 13 | 10 | 9 | 7 | 5 | 7 | 8 | 7 | 7 | 8 | 7 | 7 |
| Mouth Coating | 10 | 8 | 9 | 8 | 12 | 7 | 10 | 11 | 25 | 10 | 9 | 8 | 10 | 9 | 7 | 9 |
| Salty | 5 | 7 | 14 | 7 | 8 | 4 | 8 | 9 | 3 | 3 | 3 | 4 | 4 | 1 | 17 | 16 |
| Slippery/Slick | 11 | 8 | 9 | 6 | 11 | 10 | 8 | 11 | 6 | 8 | 9 | 7 | 9 | 9 | 8 | 7 |
| Smooth | 10 | 6 | 9 | 7 | 9 | 10 | 10 | 14 | 10 | 10 | 11 | 11 | 7 | 9 | 11 | 10 |
| Sour | 14 | 11 | 4 | 17 | 2 | 3 | 4 | 5 | 5 | 3 | 3 | 9 | 5 | 4 | 3 | 34 |
| Sweet | 15 | 15 | 14 | 2 | 14 | 23 | 4 | 3 | 7 | 35 | 6 | 18 | 2 | 3 | 4 | 4 |
| Thick | 23 | 6 | 5 | 4 | 11 | 4 | 7 | 4 | 14 | 8 | 7 | 6 | 12 | 5 | 5 | 6 |
| Thin | 5 | 7 | 6 | 5 | 5 | 10 | 9 | 4 | 5 | 6 | 10 | 29 | 5 | 11 | 9 | 21 |

Supplementary Table 5: LMM overall summary and variance components for taste intensity.

|  | **Fixed Effects** | | | | | | | | |  |
| --- | --- | --- | --- | --- | --- | --- | --- | --- | --- | --- |
|  | | |  | Sweetness (Sucrose) | | | Sourness  (CA) | | Saltiness  (NaCl) | |
| Intercept (β₀) | | |  | 58.03±3.11 | | | 59.75 ±-3.2 | | 57.83±3.01 | |
| Viscosity (β₁) | | |  | -3.43±0.86 | | | -3.29 ± 0.95 | | -4.16±1.11 | |
|  | **Random Effects** | | | | | | | | |  |
|  | |  | | | Sweetness (Sucrose) | Sourness  (CA) | | Saltiness  (NaCl) | |  |
| Panelist Variance | |  | | | 444.5 | 419.4 | | 352.7 | |  |
| Residual Variance | |  | | | 145.9 | 269.1 | | 242.7 | |  |
|  | **Model Fit** | | | | | | | | |  |
|  | |  | | | Sweetness (Sucrose) | Sourness  (CA) | | Saltiness  (NaCl) | |  |
| Conditional R² | |  | | | 0.757 | 0.617 | | 0.603 | |  |
| Marginal R² | |  | | | 0.017 | 0.021 | | 0.025 | |  |
| AIC | |  | | | 1896.76 | 2000.03 | | 1973.4 | |  |

Supplementary Table 6: Pairwise comparisons of taste intensity across thickener levels.

| **Tastant** | **Comparison to NT** | **Est.** | **SE** | **t-ratio** | **p-value** |
| --- | --- | --- | --- | --- | --- |
| NaCl | 0.80T | -6.68 | 1.78 | -3.76 | <0.001 |
|  | 1.20T | -7.93 | 2.11 | -3.76 | <0.001 |
|  | 2.60T | -10.64 | 2.83 | -3.76 | <0.001 |
| CA | 0.80T | -6.81 | 1.96 | -3.47 | 0.002 |
|  | 1.20T | -7.85 | 2.27 | -3.47 | 0.002 |
|  | 2.60T | -10.22 | 2.95 | -3.47 | 0.002 |
| Sucrose | 0.80T | -6.58 | 1.65 | -3.99 | <0.001 |
|  | 1.20T | -7.20 | 1.80 | -3.99 | <0.001 |
|  | 2.60T | -8.18 | 2.05 | -3.99 | <0.001 |

This table presents the contrasts between thickener levels (vs. NT (no-thickener)) for each tastant, adjusted using Sidak correction. Estimates represent intensity change relative to NT.

Supplementary Table 7: LMM overall summary and variance components for overall liking. Singular fit (equal panelist/residual variances) prevented the AIC calculation from being used for control (water).

|  | | **Fixed Effects** | | | | | | | | |  |
| --- | --- | --- | --- | --- | --- | --- | --- | --- | --- | --- | --- |
|  | | Water | Sucrose | |  | | CA | | NaCl | | |
| Intercept (β₀) | | -47.83±5 | -1.18±5.22 | |  | | -45.61±4.82 | | -49.07±4.94 | | |
| Log-Viscosity (β₁) | | -47.83±5 | -10.64±1.82 | |  | | -7.23±2 | | 0.47±1.44 | | |
| **Random Effects** | | | | | | | | | | |  |
|  | | Water | Sucrose |  | | CA | | NaCl | | |  |
| Panelist Variance (σ²) | | 521.1 | 1085.1 |  | | 795.8 | | 1013.9 | | |  |
| Residual Variance (σ²) | | 521.1 | 653.4 |  | | 793.9 | | 616.9 | | |  |
| **Model Fit** | | | | | | | | | | |  |
|  | | Water | Sucrose |  | | CA | | NaCl | | |  |
| Conditional R² | | 0.494 | 0.645 |  | | 0.515 | | 0.622 | | |  |
| Marginal R² | | 0.494 | 0.055 |  | | 0.028 | | 0 | | |  |
| AIC | | - | 2191.6 |  | | 2211.6 | | 2178.8 | | |  |

Supplementary Table 8: Pairwise Comparisons of Liking Rating Across Thickener Levels.

| **Tastant** | **Comparison vs NT** | **Est.** | **SE** | **t-ratio** | **p-value** |
| --- | --- | --- | --- | --- | --- |
| Water | 0.80T | -40.98 | 4.28 | -9.57 | <0.001 |
|  | 1.20T | -44.35 | 4.64 | -9.57 | <0.001 |
|  | 2.60T | -47.83 | 5.00 | -9.57 | <0.001 |
| NaCl | 0.80T | -11.62 | 3.21 | -3.61 | 0.001 |
|  | 1.20T | -13.77 | 3.81 | -3.61 | 0.001 |
|  | 2.60T | -18.49 | 5.12 | -3.61 | 0.001 |
| CA | 0.80T | 0.98 | 2.97 | 0.33 | 0.983 |
|  | 1.20T | 1.13 | 3.43 | 0.33 | 0.983 |
|  | 2.60T | 1.47 | 4.47 | 0.33 | 0.983 |
| Sucrose | 0.80T | -20.42 | 3.49 | -5.85 | <0.001 |
|  | 1.20T | -22.33 | 3.82 | -5.85 | <0.001 |
|  | 2.60T | -25.38 | 4.34 | -5.85 | <0.001 |

This table presents contrasts between thickener levels (versus NT (no thickener)) for each tastant, adjusted using Sidak correction. Estimates indicate liking rating changes relative to NT.
